# Minimum-Excess-Work Guidance: Score-Based Sampling with Experimental Data or Sparse Restraints

**DOI:** 10.1021/acs.jctc.6c00080

**Published:** 2026-05-18

**Authors:** Christopher Kolloff, Tobias Höppe, Emmanouil Angelis, Mathias Jacob Schreiner, Stefan Bauer, Andrea Dittadi, Simon Olsson

**Affiliations:** † Department of Computer Science and Engineering, 11248 Chalmers University of Technology and University of Gothenburg, SE-41296 Gothenburg, Sweden; ‡ Research Laboratory of Electronics, Massachusetts Institute of Technology, Cambridge, Massachusetts 02139, United States; § 9184 Technical University of Munich, 80333 Munich, Germany; ∥ Helmholtz AI, Helmholtz Munich, 85764 Neuherberg, Germany; ⊥ Max Planck Institute for Intelligent Systems, 72076 Tubingen, Germany

## Abstract

Surrogate models, such as Boltzmann generators (BGs) and emulators (BEs), based on deep generative models are becoming an important tool in molecular simulation. Often, we may want to use additional external information such as sparse experimental data to refine these models. However, there is no unique way to achieve this goal. Here, we propose a method inspired by thermodynamic work from statistical mechanics to regularize the guidance of pretrained probability flow generative models (e.g., continuous normalizing flows or diffusion models) to match additional sparse information. The regularization ensures that the *excess work* of the guidance procedure is minimized. We developed two guiding strategies based on this method: Path Guidance, which facilitates sampling of rare transition states by concentrating probability mass on user-defined subsets, and Observable Guidance, which aligns generated distributions with experimental observables while preserving entropy. We demonstrate the framework’s versatility on two coarse-grained Boltzmann emulators, showcasing its ability to sample transition configurations and to correct systematic biases using experimental data on a variety of model protein systems. Finally, we provide bounds on the distributional differences between the guided and unguided distributions. The method bridges thermodynamic principles with modern generative architectures, offering a principled, efficient, and physics-inspired alternative to standard fine-tuning in data-scarce domains. Our results highlight improved sample efficiency and bias reduction, underscoring their applicability to molecular simulations and beyond.

## Graphical abstract

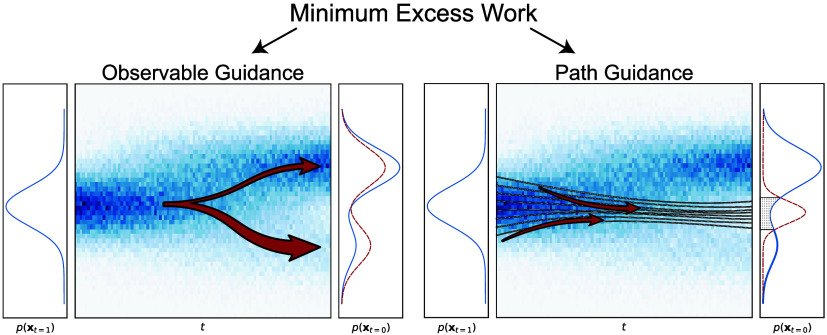

## Introduction

Probability flow generative models, such as normalizing flows^1–4^ and diffusion models,^5,6^ enable the modeling of complex, high-dimensional statistical distributions. These methods, are increasingly being used to build surrogate models for molecular dynamics such as Boltzmann Generators (BG),^7^ Boltzmann Emulators (BE),^8–10^ and implicit transfer operators (ITO),^11–13^ collectively referred to as Generative MD.^14^ These models generate samples through the integration of differential equations evolving a tractable distribution, e.g., a high-dimensional Gaussian, in a latent space, into an approximation of the data distribution. Although these models excel at general distribution learning, many scientific applications require precise control over generated samples to meet sparse observational constraints (e.g., limited transition-state configurations or partial density constraints from experiments). Current guidance methods struggle in data-scarce regimes as they typically rely on either specialized training or abundant reward signals. Existing approaches often involve fine-tuning,^15–17^ incorporating conditional information during training,^18,19^ or training an additional noise-aware discriminative model.^5^

While effective, these methods may be impractical in sparse-data regimes common in molecular and scientific applications. When the bias is large, the effective sample size of the reweighted distribution collapses, so any model retrained on the reweighted data overfits to a small number of high-weight configurations rather than the full shifted distribution. For transferable foundation models such as BioEmu, principled fine-tuning would require evaluating a KL regularizer between the pretrained and fine-tuned models at each update step—computationally prohibitive at scale and potentially causing catastrophic forgetting of the base model’s generalization. This motivates a new approach in which we apply minimal perturbations to the trained model at inference time, enabling controlled generation under very sparse constraints without modifying any model parameters. Inspired by statistical mechanics, we introduce an approach for regularizing guidance of probability flow generative models based on the principle of minimum excess work (MEW). In this context, “work” is a measure of the physical effort, e.g., energy, needed to transform a system from one macrostate to another, where a macrostate is characterized by a probability density function. MEW thereby acts as a natural, physics-inspired regularization scheme for guiding generative models. We develop the theoretical framework for MEW-based regularization of generative models, explicitly connecting it to optimal transport theory, and validate its effectiveness through extensive benchmarks across multiple scales and systems. In addition to introducing the MEW framework, we propose a simple yet effective form of path guidance tailored to sparse sampling problems. We specialize MEW guidance to two common challenges in molecular simulation. First, **Observable Guidance**: a bias-correction method that matches experimental observables while preserving the entropy of the reference ensemble via a minimum-excess-work regularizer. We validate this approach on a toy system and two coarse-grained protein Boltzmann emulators. With this approach, we thus correct the systematic bias in the base model and are therefore able to improve the prediction of unmeasured observables since they report on the same thermodynamics. Second, **Transition-State Sampling**: a path guidance-based sampling strategy that concentrates samples on user-specified regions, e.g., the low-probability transition region between states, which we evaluate on the coarse-grained Boltzmann emulator.[Fn fn1]

## Background and Theory

**Diffusion models** learn a stochastic process that maps a simple prior distribution *p*_1_ to an approximation *p*_0_ of the data distribution *q*_0_. This is typically done by reversing a known noising process governed by an Ornstein–Uhlenbeck SDE, dx_*t*_ = **f**(**x**_*t*_, *t*) d*t* + *g*(*t*) d**w**_*t*_ with **f**(**x**_*t*_, *t*) linear in **x**_*t*_. This process induces a family of marginals *q*_*t*_ with simple forward transitions $q_{t}\left(x_{t}|x_{0}\right)=N\left(x_{t};\alpha_{t}x_{0},\sigma_{t}^{2}I\right)$ , where α_*t*_ and σ_*t*_ are determined by the SDE coefficients and **x**_0_ ∼ *q*_0_(**x**) is drawn from the data distribution. Given *q*_1_ and the score ∇_**x**_*t*__ log *q*_*t*_(**x**_*t*_), one can sample from *q*_0_ via the time reversal:^20^

1 $$dx_{t}=\left[f\left(x_{t},t\right)-g{\left(t\right)}^{2}\nabla_{x_{t}}\;\log q_{t}\left(x_{t}\right)\right]\;dt+g\left(t\right)\;d{\mathrm{w̃}}_{t},,x_{1}∼q_{1}$$

where **w̃**_*t*_ is a reverse-time Wiener process or via the *probability flow ODE*:^5,21^

2 $$\frac{dx_{t}}{dt}=f\left(x_{t},t\right)-\frac{1}{2}g{\left(t\right)}^{2}\nabla_{x_{t}}\;\log q_{t}\left(x_{t}\right),,x_{1}∼q_{1}$$

both having the same time-marginals *q*_*t*_ as the forward process. In practice, the score is approximated by a score model s_θ_(**x**_*t*_, *t*), and a simple distribution *p*_1_ ≈ *q*_1_ is used as initial distribution at *t* = 1:

3 $$dx_{t}=\left[f\left(x_{t},t\right)-g{\left(t\right)}^{2}s_{\theta }\left(x_{t},t\right)\right]\;dt+g\left(t\right)\;d{\mathrm{w̃}}_{t},,x_{1}∼p_{1}$$

4 $$\frac{dx_{t}}{dt}=f\left(x_{t},t\right)-\frac{1}{2}g{\left(t\right)}^{2}s_{\theta }\left(x_{t},t\right),,x_{1}∼p_{1}$$

We denote by {*p*_*t*_}_*t*∈[0,1]_ the probability path induced by eqs 3 or 4.

### Equilibrium Sampling of the Boltzmann Distribution

A key challenge in statistical mechanics is to generate independent samples from the Boltzmann distribution

5 x∼p(x)∝exp[−βU(x)]

where β = (*k*_B_ *T*)^−1^ is the inverse temperature and *U*(**x**) is the potential energy of a configuration $x\in \Omega \subseteq R^{d}$ . This distribution underlies the estimation of macroscopic observables, such as $E_{p\left(x\right)}\left[O_{i}\left(x\right)\right]$ , which allow for a direct comparison to experimental data. However, sampling from *p*(**x**) is notoriously difficult due to the rugged energy landscape *U*(**x**). Traditional methods such as Molecular Dynamics (MD) or Markov Chain Monte Carlo (MCMC) suffer from slow mixing and generate highly correlated samples that often fail to cross energy barriers between metastable states. This leads to biased estimates and poor coverage of transition configurations, i.e., regions in state space that are severely undersampled but mechanistically crucial. Recent work on Boltzmann Generators^7,22–26^ addressed these challenges by learning direct mappings from simple priors to Boltzmann-like distributions. However, because these models are trained on data generated under a fixed potential energy function *U*(**x**), any inaccuracy in *U* propagates into the learned distribution.^27,28^ When experimental measurements are available that reveal such discrepancies, the question arises how to correct the generative model *post hoc* using sparse observational constraints. Separately, for applications such as transition-path sampling or mechanistic studies of folding, one often requires enhanced sampling of transition regions that are rare under the Boltzmann distribution. This is not due to the model being wrong but because the target application demands a different sampling distribution. In this work, we address both challenges by guiding a generative model using sparse experimental or structural information, leveraging a coarse-grained Boltzmann emulator inspired by Arts et al.^29^ and show how our method can be integrated into a state-of-the-art Boltzmann emulator^10^ to sample protein ensembles consistent with experimental data.

**Maximum Entropy Reweighting** is a broadly adopted technique to overcome force-field inaccuracies in potential energy models.^30–38^ The result of this optimization is a tilted distribution which depends on a set of Lagrange multipliers, {λ_*i*_}, each corresponding to an experimental observable of interest. The solution *p*′(**x**) ∝ *p*(**x**) exp­(−∑_*i* = 1_^*M*^ λ_*i*_ *O*_*i*_(**x**)) minimizes the KL divergence from the reference distribution *p*(**x**), subject to the constraints $E_{p\prime \left(x\right)}\left[O_{i}\left(x\right)\right]=o_{i}$ . A detailed derivation is provided in Supporting Information A.1 for the reader’s convenience. However, this approach has two limitations in the generative setting. First, it operates on a fixed set of samples $X={\left\{x_{i}\right\}}_{i=1}^{M}$ . When the bias in *p*(**x**) is substantial, the effective sample size collapses and the tilted distribution *p*′(**x**) is represented by only a small fraction of high-weight configurations. Second, one cannot simply retrain a new generative model on this tilted distribution to recover *p*′(**x**). The retrained model would overfit to those same high-weight samples rather than learn the full shifted distribution, thus inheriting the degeneracy problem. These considerations motivate applying the maximum entropy principle directly within the generative process rather than as a *post hoc* or retraining step.

**Loss Guidance** is the process of adjusting the diffusion process to satisfy a target condition **y** without fine-tuning and has been explored in several prior works.^39–41^ To sample from the conditional distribution *p*(**x**_0_|**y**) *post hoc*, we can use the following identity: ∇_**x**_*t*__ log *p*(**x**_*t*_|**y**) = ∇_**x**_*t*__ log *p*(**x**_*t*_) + ∇_**x**_*t*__ log *p*(**y**|**x**_*t*_). Obtaining ∇_**x**_*t*__ log *p*(**y**|**x**_*t*_) typically requires training a separate model on the noisy states **x**_*t*_, as done in classifier guidance.^5^ Alternatively, the posterior mean ${\mathrm{x̂}}_{t}\left(x_{t}\right)≔E_{p\left(x_{0}|x_{t}\right)}\left[x_{0}\right]$ can be used as an estimate of the clean data **x**_0_. Using Tweedie’s formula, the posterior mean can be expressed as $E_{p\left(x_{0}|x_{t}\right)}\left[x_{0}\right]=\frac{1}{\alpha_{t}}\left[x_{t}+{\sigma_{t}}^{2}\nabla_{x_{t}}\;\log p\left(x_{t}\right)\right]$ . This allows us to approximate the likelihood in data space via $\log p\left(y|{\mathrm{x̂}}_{t}\left(x_{t}\right)\right)\approx l\left({\mathrm{x̂}}_{t}\left(x_{t}\right),y\right)$ , where l denotes a suitable differentiable loss function (e.g., cross-entropy or log-likelihood under a differentiable model). The gradient ∇_**x**_*t*__ log *p*(**y**|**x̂**_*t*_(**x**_*t*_)) can then be computed by backpropagation. In practice, the mean is approximated using the score model s_θ_(**x**_*t*_, *t*), allowing the score estimate to be updated as $\nabla_{x_{t}}\;\log p\left(x_{t}|y\right)\approx s_{\theta }\left(x_{t},t\right)+\eta_{t}\nabla_{x_{t}}l\left({\mathrm{x̂}}_{t}\left(x_{t}\right),y\right)$ with η_*t*_ being a guiding strength function. We note that this procedure is a heurisitc approximation to exact posterior sampling. Replacing the posterior by a delta function incurs an approximation error that is severe at large *t* where the posterior is broad.^40,41^ This limitation motivates our path guidance formulation, which avoids backpropagation through the posterior mean.

### Work and Optimal Transport

In statistical mechanics, thermodynamic work *W* is the energy required to transform a system from a probabilistic state *p* to another *p*′. For a continuum system:

6 W=∬J(x,t)·F(x,t)dxdt

where **J**(**x**, *t*) = **v**(**x**, *t*)*p*_*t*_(**x**) is the probability flux and **F**(**x**, *t*) is the force applied to the system. This generalizes the classical work expression *W* = ∫*F*(**x**)­dx.^42^ When the force and velocity field coincide (i.e., the Jacobian of the push-forward map associated with the velocity field is a diffeomorphism), they can be expressed as spatial gradients of a potential *u*(**x**, *t*).^43^ Under these conditions, *W* becomes equivalent to the kinetic energy in the Benamou–Brenier formulation of optimal transport^44^ and provides an upper bound on the squared 2-Wasserstein distance between the distributions:

7 $$W_{2}^{2}\left(p,p^{\prime }\right)\leq \iint {∥v\left(x,t\right)∥}^{2}p_{t}\left(x\right)\;dx\;dt=W$$

where **v** and *p* satisfy $\frac{\partial }{\partial t}p_{t}\left(x\right)=-\nabla_{x}\cdot \left[p_{t}\left(x\right)v\left(x,t\right)\right]$ . Minimizing *W* (the kinetic energy) yields the optimal transport map that transforms *p* into *p*′ along the path requiring minimal energy; by eq 7, this simultaneously tightens the upper bound on *W*_2_^2^(*p*, *p*′). The idea of identifying probability paths minimizing the kinetic energy, or more generally a Lagrangian, has recently been applied to improve the efficiency of probability flow generative models.^45–52^

## Minimum-Excess-Work Guidance

During the generative process, we transform a simple distribution $p_{1}∼N\left(0,I\right)$ into a complex data distribution *p*_0_ with support $\Omega \subseteq R^{d}$ by solving the reverse-time SDE ([Disp-formula eq3]) or the ODE ([Disp-formula eq4]). To incorporate additional constraints and align the generative process with new information, we modify the drift of eqs 3 and 4 by introducing an additive perturbation to the score model:

8 $$dx_{t}=\left(f\left(x_{t},t\right)-g{\left(t\right)}^{2}\left[s_{\theta }\left(x_{t},t\right)+h_{\nu }\left(x_{t},t\right)\right]\right)\;dt+g\left(t\right)d{\mathrm{w̃}}_{t},,x_{1}∼p_{1}$$

9 $$\frac{dx_{t}}{dt}=f\left(x_{t},t\right)-\frac{1}{2}g{\left(t\right)}^{2}\left[s_{\theta }\left(x_{t},t\right)+h_{\nu }\left(x_{t},t\right)\right],,x_{1}∼p_{1}$$

where $h_{\nu }:R^{d}\times \left[0,1\right]\to R^{d}$ is a time-dependent vector field.

**The aim of **ME**W guidance** is to satisfy a guidance objective for the guided distribution *p*_0_^′^ ≠ *p*_0_, while minimizing the *excess work* associated with **h**_ν_(**x**_*t*_, *t*), required to modify the probability density, *p*_0_. We define the excess work in the context of an unperturbed and perturbed system described by the following ODEs over *t* ∈ [0, 1] with *p*_1_ = *p*_1_^′^:

10 $$\frac{dx_{t}}{dt}=v\left(x_{t},t\right),,\frac{dx_{t}}{dt}=v\left(x_{t},t\right)+u\left(x_{t},t\right)$$

with the respective time-marginal densities *p*_*t*_, *p*_*t*_^′^. Loosely following eq 7, we define the excess work as Δ*W* ≔ ∬ ∥**u**(**x**, *t*)∥^2^ *p*_*t*_^′^(**x**) dx d*t*. For the ODEs ([Disp-formula eq4]) and ([Disp-formula eq9]), the perturbation velocity is $u\left(x,t\right)=-\frac{1}{2}g{\left(t\right)}^{2}h_{\nu }\left(x,t\right)$ , so that ${∥u∥}^{2}=\frac{g{\left(t\right)}^{4}}{4}{∥h_{\nu }∥}^{2}$ , and the excess work becomes

11 $$\Delta W\left(\vartheta \right)=\iint \frac{g{\left(t\right)}^{4}}{4}{∥h_{\nu }\left(x,t\right)∥}^{2}p_{t}^{\prime }\left(x\right)\;dx\;dt$$

To justify our choice of excess work as a regularizer, it is helpful to understand how perturbations affect the generated distribution. In particular, we would like *p*_0_^′^ to remain close to the reference base distribution *p*_0_. While stability bounds of this type have appeared in the literature on ODEs and SDEs, we restate tailored versions here for completeness, with proofs in Supporting Information A.2 and A.3.

**Proposition 1:** *Let pt and p*_*t*_^′^ *be the distributions at time t obtained by solving the ODEs* ([Disp-formula eq4]) *and* ([Disp-formula eq9]) *backward in time from the same initial distribution p*_1_ *at t =* 1. *Assume that the vector fields are measurable in time and L*_*t*_-*Lipschitz in space with L*_*t*_ *integrable. Then*,

12 $$W_{2}^{2}\left(p_{0},p_{0}^{\prime }\right)\leq \int_{0}^{1}w_{W}\left(t\right)\frac{gt^{4}}{4}E_{x∼p_{t}^{\prime }}\left[{∥h_{\vartheta }\left(x,t\right)∥}^{2}\right]\;dt,,w_{W}\left(t\right)≔e^{t+2\int_{0}^{t}L_{s}\;ds}$$

The weight *w*_W_(*t*) arises from a Grönwall argument on ∥**x**_*t*_–**x**_*t*_^′^∥. The factor e^*t*^ comes from decoupling perturbation and displacement via Young’s inequality, and e^2∫_0_^*t*^ *L*_*s*_ d*s*^ from the Lipschitz continuity of the base vector field.

**Proposition 2:** *Let p_t_ and p*_*t*_^′^ *be the distributions at time t induced by the reverse-time SDEs* ([Disp-formula eq3]) *and* ([Disp-formula eq8]) *starting from the same distribution p*_1_ *at t* = 1. *Assume that both SDEs admit strong solutions and that* $P^{\prime }\ll P$ , *where* $P,P^{\prime }$ *are the path measures induced by the SDEs on* $C\left(\left[0,1\right],R^{d}\right)$ . *Then*,

13 $$D_{\mathrm{KL}}\left(p_{0}^{\prime }∥p_{0}\right)\leq \int_{0}^{1}w_{\mathrm{KL}}\left(t\right)\frac{gt^{4}}{4}E_{x∼p_{t}^{\prime }}\left[{∥h_{\vartheta }\left(x,t\right)∥}^{2}\right]\;dt,,w_{\mathrm{KL}}\left(t\right)≔\frac{2}{g{\left(t\right)}^{2}}$$

The weight *w*_KL_(*t*) follows from Girsanov’s theorem. The KL between path measures introduces a factor $\frac{1}{g{\left(t\right)}^{2}}$ , which, after substituting the drift difference, yields $w_{\mathrm{KL}}\left(t\right)=\frac{2}{g{\left(t\right)}^{2}}$ .

Since both bounds—for the KL divergence and the Wasserstein distance—are time-reweighted versions of the excess work Δ*W* ([Disp-formula eq11]), it serves as a natural choice of regularizer for guidance objectives.

We then optimize the parameters ϑ of the perturbation **h**_ν_ by minimizing the following:

14 $$L\left(\vartheta \right)=L_{1}\left(\vartheta \right)+\gamma \Delta W\left(\vartheta \right)$$

where $L_{1}\left(\vartheta \right)$ is a guidance objective and γ controls the regularization strength.

We now explore how this minimum-excess-work principle is applied in the two settings: (1) guidance based on expectations of observables and (2) targeted guidance toward a user-defined subspace.

### Observable Guidance

In this section, we guide a diffusion model to align with data that reflects an expectation, using the MEW approach. Using a set of Lagrange multipliers Λ = {λ_1_, ..., λ_*M*_} pre-estimated using, e.g., the algorithm outlined in Bottaro et al.,^53^ we dynamically adjust the score by estimating an augmentation factor **h**_ϑ_ that ensures $\left|E_{p\prime \left(x\right)}\left[O_{i}\left(x\right)\right]-o_{i}\right|\leq \varepsilon$ . It is noted that traditional reweighting techniques typically apply bias *post hoc*, whereas our method adapts the *generative* process. We express the guidance factor as,

15 $$h_{\nu }\left(x_{t},t\right)=-\eta_{t}\left(\vartheta \right)\sum_{i=1}^{M}\lambda_{i}\nabla_{x_{t}}O_{i}\left({\mathrm{x̂}}_{t}\left(x_{t}\right)\right)$$

In the same way that a score model s_θ_(**x**_*t*_, *t*) approximates the gradient of the log probability, **h**_ν_(**x**_*t*_, *t*) represents the gradient of the observable function with respect to the latent variable **x**_*t*_. The coefficients λ_*i*_ steers the flow toward (or away from) directions favored by the experimental observable expectation, thus “adjusting” the score of the original model. The expression in eq 15 thus reflects the maximum entropy principle applied in a generative setting. Its amplitude is modulated by η_*t*_(ϑ) = η_init_ exp­(−κ­(1–*t*)), and our optimization strategy consists of learning the parameters ϑ = {η_init_, κ} of this scalar function. It is noted that we use the mean posterior estimation **x̂**_*t*_(**x**_*t*_) discussed in the background section, instead of using **x**_*t*_ directly.^39,40^ Our optimization objective is 2-fold: we aim to reduce the discrepancies between the model predictions and experimental data while minimizing the excess work exerted by the augmentation. The former is a supervised loss defined as

16 $$L_{1}\left(\vartheta \right)=\frac{1}{M}\sum_{i=1}^{M}{\left(o_{i}^{\exp}-E_{x∼p_{0}^{\prime }}\left[O_{i}\left(x\right)\right]\right)}^{2}$$

where *o*_*i*_^exp^ denotes the experimental values and $E_{x∼p_{0}^{\prime }}\left[O_{i}\left(x\right)\right]$ denotes the expected values under the adjusted distribution *p*_0_^′^. To balance accuracy with the principle of maximum entropy, we introduce a regularization term based on minimizing the excess work Δ*W*. Substituting the specific form of **h**_ν_(**x**_*t*_, *t*) from eq 15 into eq 11, we obtain

17 $$\Delta W\left(\vartheta \right)=\int_{0}^{1}\frac{g{\left(t\right)}^{4}}{4}|\eta_{t}\left(\vartheta \right){|}^{2}E_{x∼p_{t}^{\prime }}\left[{∥\sum_{i=1}^{M}\lambda_{i}\nabla_{x}O_{i}\left({\mathrm{x̂}}_{t}\left(x\right)\right)∥}^{2}\right]\;dt$$

### Path Guidance

In this setting, we assume access to a set of guiding samples $X^{g}={\left\{x^{i}\right\}}_{i=1}^{M}$ , each belonging to a target subset *A* ⊂ Ω of the sampling space. Assuming that *A* forms a coherent region rather than being scattered across distinct modes, we modify the score of the diffusion model to sample from a perturbed distribution *p*_0_^′^ that maximizes the probability mass placed in *A*. The objective does not prescribe a specific target density over *A*, but rather, the internal structure of *p*_0_^′^ within *A* is shaped implicitly by the MEW regularizer, which penalizes unnecessary deviations from the base model, effectively encouraging the guided distribution to approximate the conditional Boltzmann $\frac{I_{\left\{x\in A\right\}}p_{0}\left(x\right)}{Z_{A}}$ . Training an emulator directly on *A* would be impractical in our setting, since we have access to only very few samples. Since $L_{1}$ does not need to be differentiable, the objective can be formulated generally as

18 $$L_{1}\left(\vartheta ,\varphi \right)=1-E_{x∼p_{0}^{\prime }}\left[I_{\left\{x\in A\right\}}\right]$$

That is, $L_{1}$ is the complement of the probability of drawing a sample within the target subset *A* under the guided distribution *p*_0_^′^. Guiding the diffusion process toward the subset *A* can be done by taking advantage of the probability flow ODE ([Disp-formula eq4]), which holds the desirable property of providing unique *latent representations* of each data point, for any time step *t*. Starting from the guiding samples, we compute their trajectories by integrating eq 4 forward in time, obtaining the latent representations $X_{t}^{g}={\left\{x_{t}^{i}\right\}}_{i=1}^{M}$ for time *t*. The set ${\left\{X_{t}^{g}\right\}}_{t=0}^{1}$ defines a trajectory of latent representations that the model must follow to ensure its samples satisfy **x**′ ∈ *A*. Based on this trajectory, we can define the augmentation factor as

19 $$h_{\nu ,\varphi }\left(x_{t},t\right)≔\eta_{t}\left(\vartheta \right)\nabla_{x_{t}}\;\log K_{h_{t}\left(\varphi \right)}\left(x_{t},X_{t}^{g}\right)$$

with $K_{h_{t}\left(\varphi \right)}\left(x_{t},X_{t}^{g}\right)≔\sum_{x_{t}^{i}\in X_{t}^{g}}K_{h_{t}\left(\varphi \right)}\left(x_{t},x_{t}^{i}\right)$ , where K can be any differentiable kernel with time-dependent bandwidth *h*_*t*_(φ). By updating the score function using eq 19, we align the sampling trajectory with that of the guiding points, while regularizing the guidance strength via the same excess work penalty as in eq 17, now evaluated using the time-dependent KDE score $E_{x∼p_{t}^{\prime }}\left[{∥\nabla_{x}\;\log K_{h_{t}\left(\varphi \right)}\left(x,X_{t}^{g}\right)∥}^{2}\right]$ . In practice, both η_*t*_(ϑ) and *h*_*t*_(φ) are implemented as sigmoid functions with learnable parameters ϑ = (ϑ_init_, ϑ_*g*_, ϑ_*s*_) and φ = (φ_init_, φ_*g*_, φ_*s*_) (see Supporting Information B.4) and optimized for eq 14 using Bayesian optimization with Gaussian processes. The use of sigmoids allows the guidance to be stronger early in the trajectory, when **x**_*t*_ is close to the Gaussian prior, and the kernel signal is more stable, and weaker near *t* = 0, where the data distribution is more complex and direct guidance is less reliable.

## Results

We now demonstrate the application of MEW guidance across several experimental setups. We first evaluate path and observable guidance on two toy setups and then proceed to showcase our approach on a coarse-grained protein Boltzmann Emulator.

### Observable Guidance

#### Synthetic Data

We chose a fully controlled synthetic system to test MEW guidance. We set up a biased 1D quadruple-well diffusion model with an accessible ground-truth Boltzmann distribution^54^ using only the expectation of a known observable (a four-component GMM) and injecting the corresponding Lagrange multiplier via eq 8 following Bottaro et al.^53^ This simple test system displays two closely related properties in molecular dynamics: multimodality and metastability (the former being the statistical signature of the latter), while keeping the corresponding Boltzmann distribution is numerically accessible, allowing us to directly gauge our methods’ ability to recover the unbiased distribution and to unambiguously test whether guidance alone corrects distributional bias: we observe a 10-fold reduction in KL­(*p*_GT_∥*p*_M_), where *p*_GT_ is the unbiased ground-truth density and *p*_M_ is either the biased reference or the guided model, (from 0.13 to 0.019 ± 0.002; see Supporting Information Table 2 for details) while matching the observable. We note that in this controlled toy setting, where densities are available analytically and ESS collapse is not severe, retraining a model directly on *p*′(**x**) would likely yield comparable or better results. The decisive advantages of guidance over retraining arise in realistic scenarios (large systems, transferable foundation models, ESS collapse, and data scarcity) as discussed above. We also find MEW regularization is critical to prevent mode collapse and preserve distributional fidelity. See Supporting Information Figure 2 and Table 2 for overlays and metrics; ablation experiments are reported in Supporting Information Figure 10 and Table 3.

#### Coarse-Grained Protein Boltzmann Emulator (cgBE): Chignolin

To evaluate our method on a real-world task, we apply observable guidance to guide a pretrained cgBE to sample conformations of chignolin, a ten-residue mini-protein that serves as a standard benchmark in protein folding studies.^55–57^ The base model is a coarse-grained Boltzmann Emulator trained on MD simulation data of chignolin (architecture and training details in Supporting Information B.1 and B.3). All guidance is applied at inference time, and no model parameters are modified. Our task is to correct systematic biases in the equilibrium sampling using only experimental measurements while preserving physical validity. This is a challenging task given the high-dimensional structured space and unknown ground-truth distribution.

##### Experimental Setup

We use folding free energy $\Delta G=-k_{B}T\;\log\left(\frac{p_{\mathrm{folded}}}{p_{\mathrm{unfolded}}}\right)$ as our observable, which captures the relative stability of different protein conformations. The reference model *p*_MD_ shows significant bias in this metric (−1.27 kcal/mol vs experimental value of −1.87 kcal/mol^55^), making it a suitable test case. Model architecture and training details are provided in Supporting Information B.3.

##### Evaluation

Our guided model achieves substantial improvements across several metrics (see Table 1) while maintaining physical validity, which we verified through the analysis of bond lengths and torsion angles (Figures S3 to S5, in the Supporting Information). The guided model’s folding free energy (−1.82 ± 0.01 kcal/mol) closely matches the target experimental value (−1.87 kcal/mol), reducing mean squared error by an order of magnitude from 0.6 to 0.05 kcal/mol. Additionally, the KL divergence from the reference MD trajectory improves from 0.329 to 0.005 ± 0.002, demonstrating better conservation of the properties of the reference distribution, including multimodality and entropy. Figure 1 visualizes these improvements.

**Table 1:** Quantitative Metrics Evaluating the Guidance Process: Expected Observables and KL Divergence

model M	$E_{p_{M}\left(x\right)}\left[O\left(x\right)\right]$ (kcal/mol)	KL (*p*_MD_^′^∥ $p_{M}$ )
experimental	–1.87	
reference	–1.27	0.329
guided	–1.82 ± 0.01	0.005 ± 0.002

**Figure 1:**
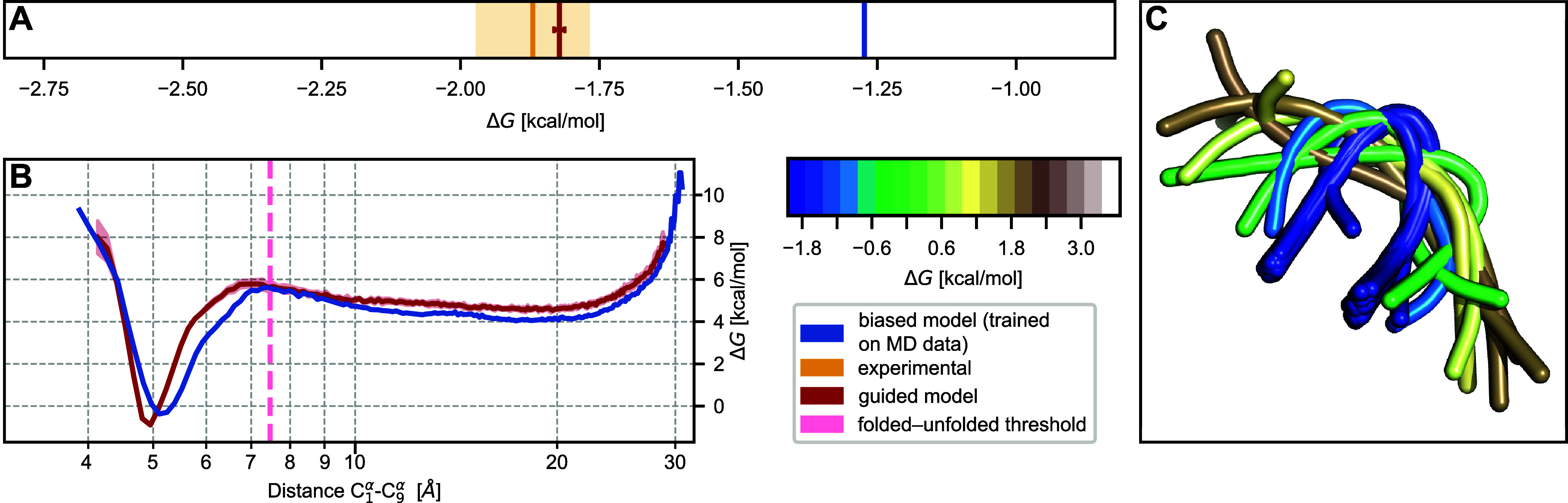
Observable guidance of chignolin. (A) Folding free energy comparison between reference model (blue), experimental data (yellow), and guided model (red). (B) Free energy profiles as a function of N- to C-terminal C^α^ distance. (C) Ensemble of 50 generated protein structures colored by their energy.

Panels A and B demonstrate that guidance successfully increases the population of folded states (*d*(C_1_^α^, C_9_^α^) < 7.5 Å), consistent with experimental observations. Panel C shows 50 superimposed generated structures, highlighting both the diversity and physical validity of our samples.

#### BioEmu: Homeodomain

Finally, we showcase our approach on the fast-folding homeodomain EnHD HTH fragment (44 residues) using BioEmu,^10^ a transferable coarse-grained Boltzmann Emulator pretrained on MD data across a large set of protein systems, and a canonical model extensively studied experimentally^58,59^ and computationally.^10,60^ As with the chignolin experiments, BioEmu serves as the unconditional base model and is held fixed throughout; guidance adjusts only the drift of the reverse-time SDE at the inference time.

##### Experimental Setup

We generate ensembles with BioEmu and compare expected ^3^ *J*_HN‑HA_ couplings to experiment. These couplings report on backbone dihedrals and, thus, thermodynamic populations. In our experiments, we use the 10 most informative observables (details in Supporting Information B.3). We quantified the agreement between experiments and computational predictions by the Q-factor^61^ and noticed that the unguided model shows a clear discrepancy (*Q* = 0.147), motivating the use of our approach. Training details are in Supporting Information B.3.

##### Evaluation

MEW markedly improves the agreement while preserving physical plausibility (Figure 2). Using only 10 experimental expectations, *Q* drops from 0.147 (unguided) to 0.037 (MEW); post hoc reweighting achieves 0.031 but with moderate weight degeneracy (relative ESS = 0.255), meaning fewer than 26% of samples contribute effectively. MEW avoids this importance-weight collapse by updating the generative process directly. Retraining BioEmu on the reweighted samples would face the same degeneracy. The model would overfit to high-weight configurations rather than learn the full shifted distribution, and updating BioEmu’s full parameter set would be computationally prohibitive without dedicated infrastructure. After guidance, points cluster near the identity within experimental uncertainty; the inset ensemble shows broad conformational coverage without mode collapse, and a representative observable (residue 38) shifts toward the experimental region without variance loss. Full histograms and structural diagnostics are in Supporting Information Figures S8 and S9. Overall, MEW uses sparse 1D NMR readouts to make targeted, physically consistent adjustments to the sampling of EnHD using BioEmu.

**Figure 2:**
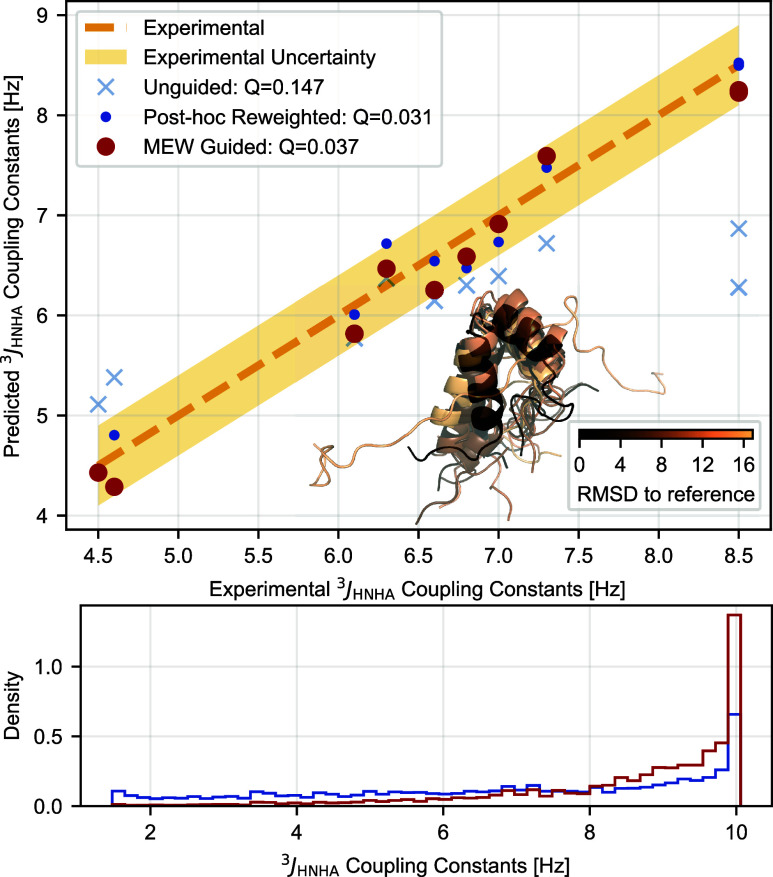
Observable guidance on EnHD. Top: 10 experimental ^3^ *J* vs unguided (light blue), MEW-guided (red), and post hoc reweighted predictions; inset: 10 sampled structures colored by RMSD. Bottom: representative histogram of residue 38 illustrating the guided population shift; blue (dashed) shows the unguided BioEmu predictions, red (solid) shows the MEW-guided predictions.

These results demonstrate that guidance in the sparse-data regime with MEW regularization allows us to effectively align high-dimensional and highly structured generative models with experimental constraints without compromising local physical validity and maintaining global distributional properties such as multimodality.

### Path Guidance

We now use the cgBE to evaluate path guidance with MEW regularization for upsampling high-energy transition configurations (states), which are critical for understanding the folding process of proteins. Due to their high energy (eq 5), these states account for only 1% of both the data and model distribution, making their successful upsampling a strong demonstration of our method’s effectiveness. Consistent with our Observable Guidance section, we also use the chignolin mini-protein, to investigate the effectiveness of path guidance and will later show that we can scale our method to a 65-residue protein. To contextualize path guidance, we first introduced an alternative baseline.

#### Baseline

As a natural alternative to path guidance, we adapt loss guidance to our setting by using the log-likelihood of a KDE fitted on guiding points $X_{0}^{g}={\left\{x_{0}^{i}\right\}}_{i=1}^{M}$ . Specifically, we will change the perturbation kernel from eq 19 to $K_{h_{t}\left(\varphi \right)}\left({\mathrm{x̂}}_{t}\left(x_{t}\right),X_{0}^{g}\right)$ . While it appears similar to path guidance, the key difference lies in the space in which the KDE is computed. In path guidance, the kernel is applied along the trajectory ${\left\{X_{t}^{g}\right\}}_{t=0}^{t=1}$ , resulting in a distinct KDE for each time step *t*. In contrast, loss guidance computes the KDE in data space and estimates the likelihood with respect to the posterior mean **x̂**_*t*_(**x**_*t*_), which requires backpropagating through the model at every sampling step. Implementation details and ablation studies for two alternative baselines that do not augment the vector field are provided in Supporting Information B.4 and D.3. To explore the dynamics of both methods, we design a synthetic example to study the effect of different parameters (see Supporting Information D.1 for details).

#### Evaluation Criteria

We assessed the methods using three key metrics. First, we measure guiding success as the percentage of sampled transition configurations (see Supporting Information B.2 for details). Second, we evaluate the diversity among transition states using the Vendi score (VS)^62^ to verify that our method generates novel samples rather than merely resampling the guiding data. Lastly, since we cannot evaluate the energy under the coarse-grained model, we instead ensure the physical validity of the generated samples under guidance by computing the Wasserstein distance (WD) between the bond-length distributions of generated and ground-truth samples, which quantifies how well our method preserves the local molecular structure.

#### Transition-State Sampling

For the transition configuration sampling task, we adapt the kernel to handle rigid-body transformations using the Kabsch algorithm^63^ akin to that adopted in ref 64. Since we found loss guidance to be difficult to optimize in this application, we first performed a large grid search to identify the optimal parameters for a fair comparison.This analysis revealed that increasing the guidance strength deteriorates sample quality in loss guidance, preventing it from achieving meaningful guiding success (Figure S13B in the Supporting Information). The performance gap stems from two key disadvantages of the loss guidance.

First, it requires using the posterior mean to compute the augmentation factor, which, especially at large *t*, suffers from very high variance. Second, at small *t*, while the predictions become more accurate, the KDE fails to capture the distribution of the guiding points, as it is not well-suited for high data complexity. As a result, the loss signal can degrade the sampled data, as evident from the increasing Wasserstein distance as the guiding strength ϑ_init_ increases (Figure 3A). In contrast, path guidance circumvents this issue by applying stronger guidance for larger *t*, where the latent is primarily noise, and decreasing it while sampling. Notably, in Figure 3A, we observe that both quality and diversity remain largely unaffected by the initial guiding strength ϑ_init_. We further investigate the difference between path and loss guidance in Supporting Information D.2.

**Figure 3:**
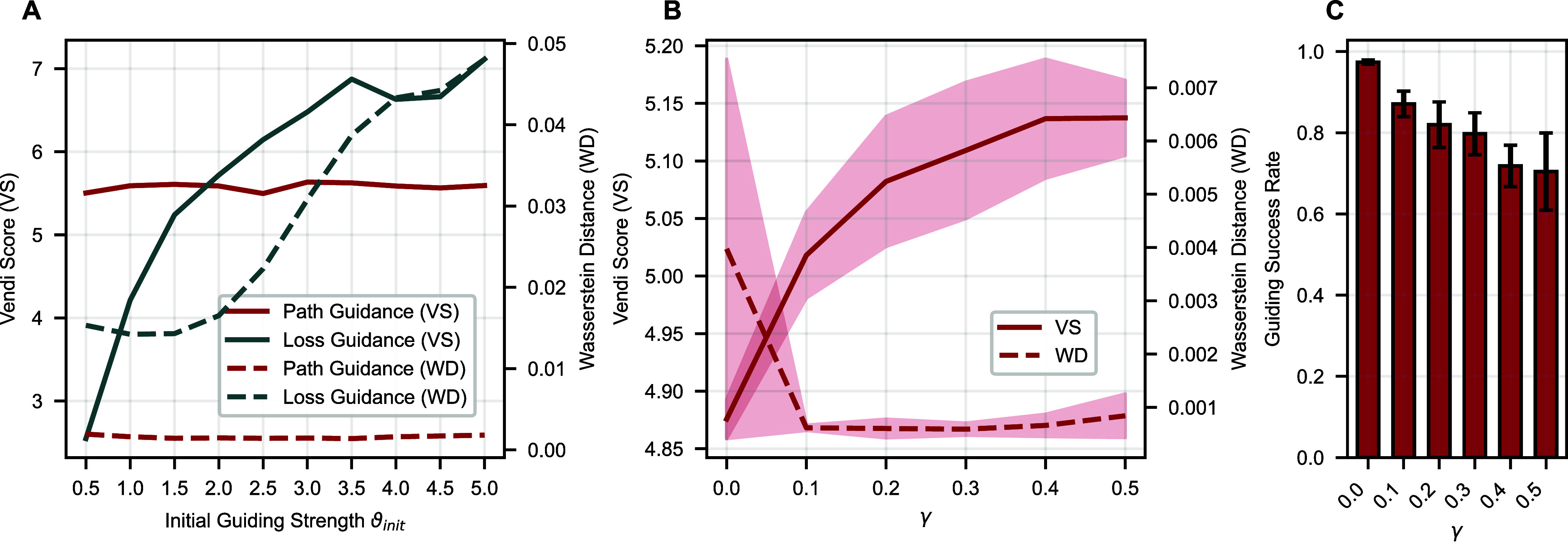
Path guidance vs loss guidance for sampling transition states. (A) Sample quality and diversity, measured by the Wasserstein Distance (WD) and Vendi score (VS), show that path guidance preserves diversity and quality even at high guiding strengths, whereas loss guidance deteriorates. (B) Without MEW regularization (γ = 0), sampled transition states tend to collapse and have no diversity (VS). Regularization also improves sample quality (WD). (C) Guiding success rate, measured as the percentage of transition states sampled, for different regularization strengths.

After observing that loss guidance could not be reliably optimized, we conducted a separate set of experiments to evaluate path guidance within the MEW framework by optimizing the objective in eq 14. Disabling regularization (γ = 0) results in the highest guidance success rates (Figure 3C) but produces highly degenerate samples and reduced structural diversity, as indicated by the large variance in Wasserstein distance. In contrast, applying MEW regularization improves both sample quality and diversity (Figure 3B), while incurring only a modest reduction in guidance success. Overall, our results demonstrate that path guidance offers a strong alternative to loss guidance and that MEW regularization is essential for robust and physically meaningful sampling in data-sparse regimes. Hence, with only minor modifications, we can scale path guidance.

To assess whether Path Guidance scales to higher-dimensional systems, we evaluate it on the 65-residue Protein G^65^ using the BioEmu cgBE.^10^ Because BioEmu represents both backbone geometry and local frames, we adapt the KDE-based guidance term to operate on the rotation tangent space (see Supporting Information B.4.3 for details). We found that standard sampling barely represents the transition states (∼0.1%), hence, already minor upsampling would be remarkably helpful. Our experiments show that path guidance with MEW regularization increases the proportion of sampled transition states to ∼28% while preserving physical validity (see Figure 4). With MEW regularization, the Wasserstein Distance (WD) between guided and reference bond-length distribution is WD = 0. 08 ± 0.002, and the Vendi score is VS = 10. 88 ± 0.33. Without regularization, sample quality degrades (WD = 0. 09 ± 0.002) while the Vendi score remains within variation (VS = 10. 94 ± 0.31), consistent with our findings on chignolin and further supporting the necessity of MEW regularization for maintaining sample quality under guidance.

**Figure 4:**
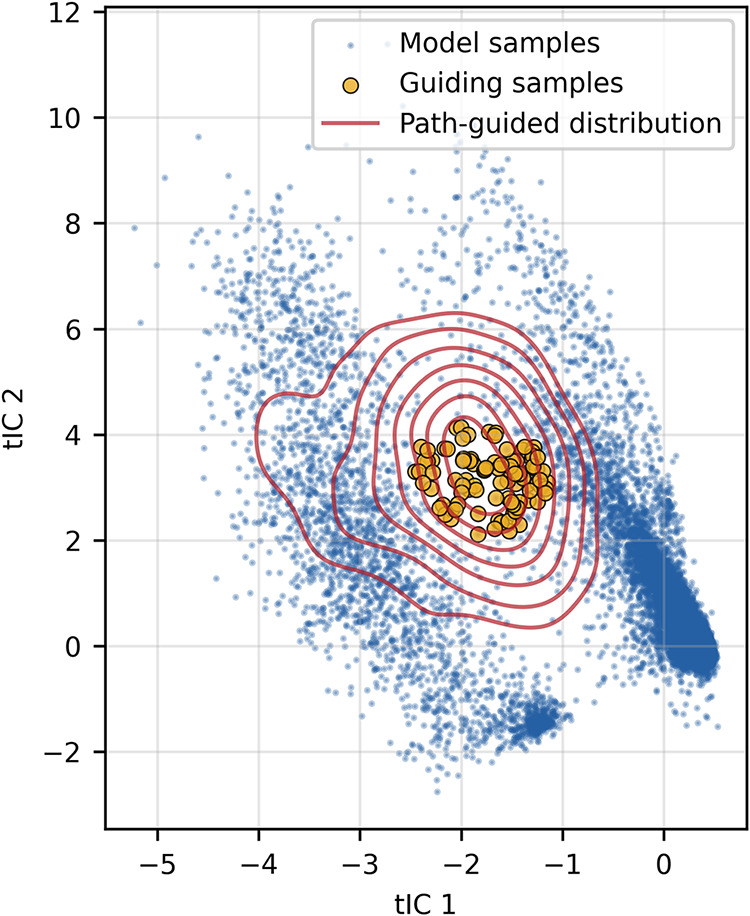
Path guidance on Protein G. Applying path guidance to Protein G increases sampling in the transition region while preserving biologically meaningful conformations. Shown is a 2D tICA projection of sampled configurations, highlighting samples under standard sampling (blue), transition-state regions used for guidance (yellow) and samples under guidance (red). The mode of the guided distribution is placed on the guidance samples and remains quantitatively consistent with the original transition-state ensemble.

## Related Works

### Stochastic Optimal Control

MEW also naturally connects to recent advances in stochastic optimal control (SOC) applied to diffusion and flow-based generative models. In particular, to approaches which consider steering generative trajectories by balancing a task-specific objective, such as aligning with experimental observables or reward models with a regularization that penalizes deviation from a pretrained base model. In those works,^17,66–68^ fine-tuning the diffusion model is framed as an SOC problem that minimizes the control effort while achieving alignment with downstream goals. Conceptually, the MEW principle plays an analogous role to the control cost in SOCs, regularizing path perturbations to preserve the prior’s structure while achieving target objectives. This connection puts MEW within the broader trend of leveraging control-theoretic principles, including KL and f-divergence regularization, to derive principled, sample-efficient, and robust fine-tuning strategies for probabilistic generative models.

### Transition Ensemble Sampling

Traditional methods such as transition-path sampling^69,70^ use Monte Carlo in trajectory space, while recent machine learning approaches^71^ employ neural networks but require extensive training data or predefined collective variables. Instead of explicit path sampling, we guide the generative process by using latent representations of known transition states. While related to recent work using Boltzmann generators,^72^ our approach directly modifies the score function during sampling rather than performing MCMC moves between paths, enabling more efficient exploration of transition regions.

### Reweighting with Experimental Data

Reweighting molecular dynamics simulations using experimental data has a long history in computational chemistry and biophysics. Theoretical work^30,31,34,73^ adopted Jaynes^74^ Maximum Entropy approach to the problem, following several early experimental studies^75–77^ based on replica-averaged simulations, giving a theoretical foundation for these approaches. This work was later complemented by probabilistic and Bayesian perspectives,^32,38,53,78^ some of which specifically focused on reweighing.^33,37,79,80^

## Limitations

Despite the strong empirical performance of MEW guidance across a range of scientific settings, several limitations merit a consideration. These primarily stem from the assumptions underpinning the method’s application, e.g., that physical observables or representative samples can be leveraged to correct expectation values or guide sampling in low-density regions. While this does not require perfect model accuracy, it does require the model to be sufficiently expressive and responsive to the guidance. If key modes are absent, then convergence to meaningful distributions may fail. Additionally, the current framework assumes differentiable observables, restricting its applicability in discrete or nondifferentiable domains.

### Computational Cost

Parameter estimation (Bayesian optimization over η_init_, κ) incurs a one-time cost of minutes for the toy system and up to a few hours for larger protein systems on a single GPU; full details are in Supporting Information B.3–B.4. Once estimated, guided sampling costs almost the same as unguided sampling, since no gradients through the score network are required. This is a higher upfront cost than post hoc reweighting, but the resulting guided generator can be reused indefinitely without maintaining a large weighted sample archive. Compared to enhanced MD sampling, MEW Path Guidance is substantially cheaper. Sampling the transition region of Protein G via unbiased MD would require simulation times on the order of the exchange time scale (∼65 μs^57^), far exceeding the few GPU-hours needed for guidance.

## Conclusion

In this work, we introduced minimum-excess-work (MEW) guidance, a physics-inspired framework for guidance of pretrained probability flow generative models with sparse external information by regularizing *excess work*. Our analysis shows that this thermodynamically motivated regularization is closely connected to upper bounds on the Wasserstein distance and the KL divergence between the reference and the guided distributions. We demonstrated the effectiveness of MEW regularization in two settings: *observable guidance* and *path guidance*. These approaches enable the alignment with sparse experimental constraints and targeted sampling in low-density regions while maintaining model flexibility. By penalizing excess work, our method reduces bias and enhances the sampling of rare, physically meaningful configurations, without degrading sample quality. Our results position MEW guidance as a principled and effective tool for bias correction and informed exploration in data-scarce scientific applications, such as the refinement of coarse-grained force fields against experimental data or the generation of starting conditions for unbiased MD or transition-path sampling.

## Supplementary Material



## Footnotes

1 Code to reproduce the result are available on the dedicated repository: https://github.com/olsson-group/minimumexcesswork/.

## Notes

Proofs of propositions, experimental details, and additional results on toy systems (PDF)

## ∇

A.D. and S.O. are joint last authors.
