# Supplementary material for: Minimum-Excess-Work Guidance: Score-Based Sampling with Experimental Data or Sparse Restraints
Source: J Chem Theory Comput. 2026 May 18;22(11):5838–48. doi: 10.1021/acs.jctc.6c00080 (PMC13255250; doi:10.1021/acs.jctc.6c00080)
Supplement: Supplementary file 1 [file ct6c00080_si_001.pdf]

Supporting Information: Minimum Excess Work Guidance: Score-Based Sampling with Experimental Data or Sparse Restraints

Christopher Kolloff,[†,‡,⊥] Tobias Höppe,[¶,§,⊥] Emmanouil Angelis,[¶,§,⊥] Mathias Jacob Schreiner,[†] Stefan Bauer,[¶,§] Andrea Dittadi,[¶,§,‖,#] and Simon Olsson[*,†,#]

†*Department of Computer Science and Engineering, Chalmers University of Technology and University of Gothenburg, SE-41296 Gothenburg, Sweden*

‡*Massachusetts Institute of Technology, Research Laboratory of Electronics, Cambridge, MA, USA*

¶*Technical University of Munich*

§*Helmholtz AI, Munich*

‖*Max Planck Institute for Intelligent Systems, Tübingen*

⊥*Contributed equally to this work.*

#*Joint last authors.*

E-mail: simonols@chalmers.se

A Proofs

A.1 Short Derivation of Maximum Entropy Reweighting of MD Trajectories using Observables

The maximum entropy approach[S1] has been widely adopted[S2–S6] to derive reweighting schemes to find a minimally biased probability distribution that satisfies experimental constraints.

Consider a reference probability distribution $p(\mathbf{x})$, e.g., an empirical distribution estimated from MD simulation data, and an unknown target distribution $p'(\mathbf{x})$ that should match experimental measurements. Following Jaynes' maximum entropy principle, we seek to minimize the KL divergence from $p(\mathbf{x})$ to $p'(\mathbf{x})$ subject to the constraint that the expectations of observables $O_i(\mathbf{x})$ under $p'(\mathbf{x})$ match their experimental values o_i. That is,

$$\min_{p'} \int p'(\mathbf{x}) \log \frac{p'(\mathbf{x})}{p(\mathbf{x})} \, \mathrm{d}\mathbf{x} \tag{1}$$

subject to:

$$\mathbb{E}_{p'(\mathbf{x})}[O_i(\mathbf{x})] = o_i \quad \text{for } i = 1, \ldots, M \tag{2}$$

$$\int p'(\mathbf{x}) \, \mathrm{d}\mathbf{x} = 1 \tag{3}$$

Using the method of Lagrange multipliers, we obtain the following objective:

$$S = -\int p'(\mathbf{x}) \log \frac{p'(\mathbf{x})}{p(\mathbf{x})} \, \mathrm{d}\mathbf{x} + \sum_{i=1}^{M} \lambda_i \left(\int p'(\mathbf{x}) O_i(\mathbf{x}) \, \mathrm{d}\mathbf{x} - o_i \right) + \mu \left(\int p'(\mathbf{x}) \, \mathrm{d}\mathbf{x} - 1 \right) \tag{4}$$

where $\{\lambda_i\}_{i=1}^{M}$ are the Lagrange multipliers for the constraints on the M observables, and μ is the multiplier for density normalization. Setting the functional derivative $\delta S / \delta p'$ to zero yields

$$-\log \frac{p'(\mathbf{x})}{p(\mathbf{x})} - 1 + \sum_{i=1}^{M} \lambda_i O_i(\mathbf{x}) + \mu = 0 \ . \tag{5}$$

Finally, solving for $p'(\mathbf{x})$ and determining μ through normalization gives

$$p'(\mathbf{x}) \propto p(\mathbf{x}) \exp\left(-\sum_{i=1}^{M} \lambda_i O_i(\mathbf{x})\right) , \tag{6}$$

where the λs are determined, e.g., following Bottaro et al. [S6], such that the constraints on the expectations are satisfied. This reweighted distribution represents the maximum entropy solution that satisfies the experimental constraints while minimizing the bias introduced relative to the reference distribution $p(\mathbf{x})$.

A.2 Bounding the Wasserstein distance

In this section, we derive an upper bound on the squared Wasserstein distance $W_2^2(p_0, p_0')$, where the distributions p_0 and p_0' are obtained by evolving a common terminal distribution $p_1 = p_1'$ backward in time according to the ODEs in Eqs.4 and 9 in main manuscript. We begin by proving a Grönwall-type lemma (see, e.g., Bressan and Piccoli [S7], Lemma 2.1.2) that will be useful to prove our result.

Lemma 1. *Let $T > 0$ and let f be an absolutely continuous function over $[0, T]$ satisfying the differential inequality*

$$\frac{\mathrm{d}}{\mathrm{d}t} f(t) \leq a(t) f(t) + b(t) \qquad \text{for a.e. } t \in [0, T], \tag{7}$$

where $a, b \in L^1([0, T])$ are integrable functions. Then, for every $t \in [0, T]$,

$$f(t) \leq \exp\left(\int_0^t a(u)\,\mathrm{d}u\right) f(0) + \int_0^t \exp\left(\int_s^t a(u)\,\mathrm{d}u\right) b(s)\,\mathrm{d}s \ . \tag{8}$$

Proof. Define the absolutely continuous function

$$\psi(t) := \exp\left(-\int_0^t a(u)\,\mathrm{d}u\right)$$

and note that $\psi(t) > 0$, $\psi(0) = 1$, and

$$\frac{\mathrm{d}}{\mathrm{d}t}\psi(t) = -a(t)\psi(t) \ .$$

Multiplying both sides of Eq. (7) by $\psi(t)$ and integrating from 0 to t, we have

$$\int_0^t \psi(s)\frac{\mathrm{d}}{\mathrm{d}s}f(s)\,\mathrm{d}s \leq \int_0^t \psi(s)a(s)f(s)\,\mathrm{d}s + \int_0^t \psi(s)b(s)\,\mathrm{d}s \tag{9}$$

$$\psi(t)f(t) - \psi(0)f(0) - \int_0^t \psi'(s)f(s)\,\mathrm{d}s \leq \int_0^t \psi(s)a(s)f(s)\,\mathrm{d}s + \int_0^t \psi(s)b(s)\,\mathrm{d}s \tag{10}$$

$$\psi(t)f(t) - f(0) + \int_0^t a(s)\psi(s)f(s)\,\mathrm{d}s \leq \int_0^t \psi(s)a(s)f(s)\,\mathrm{d}s + \int_0^t \psi(s)b(s)\,\mathrm{d}s \tag{11}$$

$$\psi(t)f(t) \leq f(0) + \int_0^t \psi(s)b(s)\,\mathrm{d}s \ . \tag{12}$$

We then divide both sides by $\psi(t)$ again to conclude:

$$f(t) \leq \frac{f(0)}{\psi(t)} + \int_0^t \frac{\psi(s)}{\psi(t)}b(s)\,\mathrm{d}s \tag{13}$$

$$= \exp\left(\int_0^t a(u)\,\mathrm{d}u\right) f(0) + \int_0^t \exp\left(\int_s^t a(u)\,\mathrm{d}u\right) b(s)\,\mathrm{d}s \tag{14}$$

□

Proposition 2. *Let $T > 0$, and let $\mathbf{v}, \mathbf{v}' : [0,T] \times \mathbb{R}^d \to \mathbb{R}^d$ be measurable in time and L_t-Lipschitz in space, with L_t integrable. Let p_0 be a probability measure on $\mathbb{R}^d$, and define p_t, p'_t as the pushforwards of p_0 under the flows of the ODEs $\frac{\mathrm{d}\mathbf{x}_t}{\mathrm{d}t} = \mathbf{v}_t(\mathbf{x}_t)$ and $\frac{\mathrm{d}\mathbf{x}'_t}{\mathrm{d}t} = \mathbf{v}'_t(\mathbf{x}'_t)$. Then for all $t \in [0,T]$,*

$$W_2^2(p_t, p'_t) \leq \int_0^t \exp\left(t - s + 2\int_s^t L_u\,\mathrm{d}u\right) \mathbb{E}_{\mathbf{x}\sim p'_s}\left[\|\mathbf{v}_s(\mathbf{x}) - \mathbf{v}'_s(\mathbf{x})\|^2\right]\,\mathrm{d}s \ . \tag{15}$$

Proof. Let ϕ_t, ϕ'_t be the flows of the ODEs, i.e., $\mathbf{x}_t = \phi_t(\mathbf{x}_0)$, $\frac{\mathrm{d}\phi_t(\mathbf{x})}{\mathrm{d}t} = \mathbf{v}_t(\phi_t(\mathbf{x}))$, and similarly

for $\mathbf{x}'$ and ϕ'_t. Define the coupling:

$$\tilde{\pi}_t \coloneqq (\phi_t, \phi'_t)_* p_0 \in \Gamma(p_t, p'_t) \ , \tag{16}$$

i.e., the pushforward of p_0 through the map $\mathbf{x} \mapsto (\phi_t(\mathbf{x}), \phi'_t(\mathbf{x}))$. By definition of 2-Wasserstein distance, we can write:

$$W_2^2(p_t, p'_t) \leq \int \|\mathbf{x} - \mathbf{x}'\|^2 \, \mathrm{d}\tilde{\pi}_t(\mathbf{x}, \mathbf{x}') = \mathbb{E}_{(\mathbf{x}_t, \mathbf{x}'_t) \sim \tilde{\pi}_t} \left[\|\mathbf{x}_t - \mathbf{x}'_t\|^2 \right] \tag{17}$$

Take any $\mathbf{x}_0, \mathbf{x}'_0 \in \mathbb{R}^d$ and let $\mathbf{x}_t = \phi_t(\mathbf{x}_0)$ and $\mathbf{x}'_t = \phi'_t(\mathbf{x}'_0)$. Then,

$$\frac{\mathrm{d}}{\mathrm{d}t}\|\mathbf{x}_t - \mathbf{x}'_t\|^2 = 2(\mathbf{x}_t - \mathbf{x}'_t) \cdot (\mathbf{v}_t(\mathbf{x}_t) - \mathbf{v}'_t(\mathbf{x}'_t)) \tag{18}$$

$$= 2(\mathbf{x}_t - \mathbf{x}'_t) \cdot (\mathbf{v}_t(\mathbf{x}_t) - \mathbf{v}_t(\mathbf{x}'_t)) + 2(\mathbf{x}_t - \mathbf{x}'_t) \cdot (\mathbf{v}_t(\mathbf{x}'_t) - \mathbf{v}'_t(\mathbf{x}'_t)) \tag{19}$$

We bound the first term using the Cauchy–Schwarz inequality and the L_t-Lipschitzness of $\mathbf{v}_t$:

$$2(\mathbf{x}_t - \mathbf{x}'_t) \cdot (\mathbf{v}_t(\mathbf{x}_t) - \mathbf{v}_t(\mathbf{x}'_t)) \leq 2\|\mathbf{x}_t - \mathbf{x}'_t\| \, \|\mathbf{v}_t(\mathbf{x}_t) - \mathbf{v}_t(\mathbf{x}'_t)\| \tag{20}$$

$$\leq 2L_t\|\mathbf{x}_t - \mathbf{x}'_t\|^2 \ . \tag{21}$$

Using $0 \leq \|\mathbf{a} - \mathbf{b}\|^2 = \|\mathbf{a}\|^2 + \|\mathbf{b}\|^2 - 2\mathbf{a} \cdot \mathbf{b}$ for the second term we have:

$$2(\mathbf{x}_t - \mathbf{x}'_t) \cdot (\mathbf{v}_t(\mathbf{x}'_t) - \mathbf{v}'_t(\mathbf{x}'_t)) \leq \|\mathbf{x}_t - \mathbf{x}'_t\|^2 + \|\mathbf{v}_t(\mathbf{x}'_t) - \mathbf{v}'_t(\mathbf{x}'_t)\|^2 \ . \tag{22}$$

Plugging these two bounds into Eq. (19), we get

$$\frac{\mathrm{d}}{\mathrm{d}t}\|\mathbf{x}_t - \mathbf{x}'_t\|^2 = 2(\mathbf{x}_t - \mathbf{x}'_t) \cdot (\mathbf{v}_t(\mathbf{x}_t) - \mathbf{v}_t(\mathbf{x}'_t)) + 2(\mathbf{x}_t - \mathbf{x}'_t) \cdot (\mathbf{v}_t(\mathbf{x}'_t) - \mathbf{v}'_t(\mathbf{x}'_t)) \tag{23}$$

$$\leq 2L_t\|\mathbf{x}_t - \mathbf{x}'_t\|^2 + \|\mathbf{x}_t - \mathbf{x}'_t\|^2 + \|\mathbf{v}_t(\mathbf{x}'_t) - \mathbf{v}'_t(\mathbf{x}'_t)\|^2 \tag{24}$$

$$= (2L_t + 1)\|\mathbf{x}_t - \mathbf{x}'_t\|^2 + \|\mathbf{v}_t(\mathbf{x}'_t) - \mathbf{v}'_t(\mathbf{x}'_t)\|^2 \ . \tag{25}$$

Finally, taking expectations on both sides w.r.t. $(\mathbf{x}_t, \mathbf{x}'_t) \sim \tilde{\pi}_t$, and exchanging expectation and derivative under standard regularity assumptions, we get:

$$\frac{\mathrm{d}}{\mathrm{d}t}\mathbb{E}\left[\|\mathbf{x}_t - \mathbf{x}'_t\|^2\right] \leq (2L_t + 1)\, \mathbb{E}\left[\|\mathbf{x}_t - \mathbf{x}'_t\|^2\right] + \mathbb{E}\left[\|\mathbf{v}_t(\mathbf{x}'_t) - \mathbf{v}'_t(\mathbf{x}'_t)\|^2\right] \ . \tag{26}$$

This inequality can be expressed as

$$\frac{\mathrm{d}f(t)}{\mathrm{d}t} \leq (2L_t + 1)f(t) + b(t) \ , \qquad f(0) = 0 \ , \tag{27}$$

with

$$f(t) \coloneqq \mathbb{E}_{(\mathbf{x}_t, \mathbf{x}'_t)\sim\tilde{\pi}_t}\left[\|\mathbf{x}_t - \mathbf{x}'_t\|^2\right] \tag{28}$$

$$b(t) \coloneqq \mathbb{E}_{\mathbf{x}'_t\sim p'_t}\left[\|\mathbf{v}_t(\mathbf{x}'_t) - \mathbf{v}'_t(\mathbf{x}'_t)\|^2\right] \ . \tag{29}$$

Applying Lemma 1 with $a(t) = (2L_t + 1)$, we get:

$$f(t) \leq \int_0^t \exp\left(\int_s^t (2L_u + 1)\,\mathrm{d}u\right) b(s)\,\mathrm{d}s \tag{30}$$

$$= \int_0^t e^{t-s} \exp\left(2\int_s^t L_u\,\mathrm{d}u\right) b(s)\,\mathrm{d}s \tag{31}$$

Since from Eq. (17) we know that $W_2^2(p_t, p'_t) \leq f(t)$, the statement follows:

$$W_2^2(p_t, p'_t) \leq \int_0^t e^{t-s} \exp\left(2\int_s^t L_u\,\mathrm{d}u\right) \mathbb{E}_{\mathbf{x}\sim p'_s}\left[\|\mathbf{v}_s(\mathbf{x}) - \mathbf{v}'_s(\mathbf{x})\|^2\right]\,\mathrm{d}s \ . \tag{32}$$

□

Although the result in the time-reversed case is straightforward as it directly follows from a time reparameterization, we state it and prove it for the sake of completeness.

Proposition 3. *Let* $\mathbf{v}, \mathbf{v}' : [0,1] \times \mathbb{R}^d \to \mathbb{R}^d$ *be measurable in time and* L_t*-Lipschitz in space, with* L_t *integrable. Let* p_0, p'_0 *be probability measures on* $\mathbb{R}^d$*, and define* p_t, p'_t *as the*

pushforwards of p_0 *under the flows of the ODEs* $\frac{\mathrm{d}\mathbf{x}_t}{\mathrm{d}t} = \mathbf{v}_t(\mathbf{x}_t)$ *and* $\frac{\mathrm{d}\mathbf{x}'_t}{\mathrm{d}t} = \mathbf{v}'_t(\mathbf{x}'_t)$*. Assume* $p_1 = p'_1$*. Then,*

$$W_2^2(p_0, p'_0) \leq \int_0^1 \exp\left(t + 2\int_0^t L_s \, \mathrm{d}s\right) \mathbb{E}_{\mathbf{x}\sim p'_t}\left[\|\mathbf{v}_t(\mathbf{x}) - \mathbf{v}'_t(\mathbf{x})\|^2\right] \mathrm{d}t \ . \tag{33}$$

Proof. Consider the time reversal transformation $s = 1 - t$. Define $\tilde{\mathbf{x}}_s \coloneqq \mathbf{x}_{1-s}$ and $\tilde{\mathbf{x}}'_s \coloneqq \mathbf{x}'_{1-s}$, where $\mathbf{x}_t$ and $\mathbf{x}'_t$ satisfy the original ODEs with vector fields $\mathbf{v}_t, \mathbf{v}'_t$, with $\mathbf{x}_t \sim p_t$, $\mathbf{x}'_t \sim p'_t$, and $p_1 = p'_1$. Differentiating the reversed processes, we get:

$$\frac{\mathrm{d}\tilde{\mathbf{x}}_s}{\mathrm{d}s} = \frac{\mathrm{d}\mathbf{x}_{1-s}}{\mathrm{d}t} \cdot \frac{\mathrm{d}t}{\mathrm{d}s} = -\mathbf{v}_{1-s}(\mathbf{x}_{1-s}) = -\mathbf{v}_{1-s}(\tilde{\mathbf{x}}_s) \tag{34}$$

and similarly for $\tilde{\mathbf{x}}'$. Thus, the reversed processes satisfy:

$$\frac{\mathrm{d}\tilde{\mathbf{x}}_s}{\mathrm{d}s} = \tilde{\mathbf{v}}_s(\tilde{\mathbf{x}}_s) \ , \quad \frac{\mathrm{d}\tilde{\mathbf{x}}'_s}{\mathrm{d}s} = \tilde{\mathbf{v}}'_s(\tilde{\mathbf{x}}'_s) \ , \tag{35}$$

where we defined the reversed velocity fields $\tilde{\mathbf{v}}_s(\mathbf{x}) \coloneqq -\mathbf{v}_{1-s}(\mathbf{x})$ and $\tilde{\mathbf{v}}'_s(\mathbf{x}) \coloneqq -\mathbf{v}'_{1-s}(\mathbf{x})$. From the definitions $\tilde{\mathbf{x}}_s \coloneqq \mathbf{x}_{1-s}$ and $\tilde{\mathbf{x}}'_s \coloneqq \mathbf{x}'_{1-s}$ it directly follows that $\tilde{p}_s = p_{1-s}$ and $\tilde{p}'_s = p'_{1-s}$. At $s = 0$, we have $\tilde{p}_0 = p_1 = p'_1 = \tilde{p}'_0$, so the reversed processes start from the same distribution.

Since $\mathbf{v}_t$ and $\mathbf{v}'_t$ are L_t-Lipschitz in space with L_t integrable, $\tilde{\mathbf{v}}_s$ and $\tilde{\mathbf{v}}'_s$ are L_{1-s}-Lipschitz. The reversed ODEs start at $s = 0$ from the same distribution ($\tilde{p}_0 = \tilde{p}'_0$) and evolve to $\tilde{p}_1 = p_0$ and $\tilde{p}'_1 = p'_0$ at $s = 1$. Applying Proposition 2, we get:

$$W_2^2(\tilde{p}_1, \tilde{p}'_1) \leq \int_0^1 \exp\left(1 - s + 2\int_s^1 L_{1-u} \, \mathrm{d}u\right) \mathbb{E}_{\mathbf{x}\sim\tilde{p}'_s}\left[\|\tilde{\mathbf{v}}_s(\mathbf{x}) - \tilde{\mathbf{v}}'_s(\mathbf{x})\|^2\right] \mathrm{d}s \ . \tag{36}$$

Substituting $\tilde{p}_s = p_{1-s}$ and $\tilde{p}'_s = p'_{1-s}$, using the definitions of $\tilde{\mathbf{v}}_t, \tilde{\mathbf{v}}'_t$, and applying a change

of variables $t = 1 - s$, we obtain the desired bound:

$$W_2^2(p_0, p_0') \leq \int_0^1 \exp\left(1 - s + 2\int_s^1 L_{1-u}\,\mathrm{d}u\right) \mathbb{E}_{\mathbf{x}\sim p_{1-s}'}\left[\|\mathbf{v}_{1-s}(\mathbf{x}) - \mathbf{v}_{1-s}'(\mathbf{x})\|^2\right] \mathrm{d}s \tag{37}$$

$$= \int_0^1 \exp\left(t + 2\int_{1-t}^1 L_{1-u}\,\mathrm{d}u\right) \mathbb{E}_{\mathbf{x}\sim p_t'}\left[\|\mathbf{v}_t(\mathbf{x}) - \mathbf{v}_t'(\mathbf{x})\|^2\right] \mathrm{d}t \tag{38}$$

$$= \int_0^1 \exp\left(t + 2\int_0^t L_s\,\mathrm{d}s\right) \mathbb{E}_{\mathbf{x}\sim p_t'}\left[\|\mathbf{v}_t(\mathbf{x}) - \mathbf{v}_t'(\mathbf{x})\|^2\right] \mathrm{d}t \ . \tag{39}$$

□

In this work, we are specifically interested in the ODEs (4) and (9) in main manuscript:

Proposition (Restatement of Proposition 1 in main manuscript). Let p_t and p_t' be the distributions at time t obtained by solving the ODEs (4) and (9) backwards in time from the same initial distribution p_1 at $t = 1$. Assume that the vector fields are measurable in time and L_t-Lipschitz in space with L_t integrable. Then:

$$W_2^2(p_0, p_0') \leq \int_0^1 w_{\mathrm{W}}(t)\, \frac{g(t)^4}{4}\, \mathbb{E}_{\mathbf{x}\sim p_t'}\left[\|\mathbf{h}_\vartheta(\mathbf{x}, t)\|^2\right] \mathrm{d}t \ , \qquad w_{\mathrm{W}}(t) := e^{t + 2\int_0^t L_s\,\mathrm{d}s} \ . \tag{40}$$

Proof. The ODEs (4) and (9) have the following vector fields:

$$\mathbf{v}_t(\mathbf{x}) = \mathbf{f}(\mathbf{x}, t) - \frac{1}{2} g(t)^2 \mathbf{s}(\mathbf{x}, t)$$
$$\mathbf{v}_t'(\mathbf{x}) = \mathbf{f}(\mathbf{x}, t) - \frac{1}{2} g(t)^2 \left(\mathbf{s}(\mathbf{x}, t) + \mathbf{h}(\mathbf{x}, t)\right) \ .$$

The result directly follows by applying Proposition 3:

$$W_2^2(p_0, p_0') \leq \int_0^1 \exp\left(t + 2\int_0^t L_s\,\mathrm{d}s\right) \frac{g(t)^4}{4} \mathbb{E}_{\mathbf{x}\sim p_t'}\left[\|\mathbf{h}_\vartheta(\mathbf{x}, t)\|^2\right] \mathrm{d}t \ . \tag{41}$$

□

A.3 Bounding the KL divergence

Proposition 4. *Let* $p, p' : \mathbb{R}^d \times [0,1] \to \mathbb{R}_{\geq 0}$ *be two probability paths over time* $t \in [0,1]$*, induced by two reverse-time SDEs:*

$$\mathrm{d}\mathbf{x}_t = \boldsymbol{\mu}_t(\mathbf{x}_t)\,\mathrm{d}t + g_t\,\mathrm{d}\widetilde{\mathbf{w}}_t\ , \qquad \mathrm{d}\mathbf{x}_t = \boldsymbol{\mu}'_t(\mathbf{x}_t)\,\mathrm{d}t + g_t\,\mathrm{d}\widetilde{\mathbf{w}}'_t \tag{42}$$

where $\widetilde{\mathbf{w}}_t, \widetilde{\mathbf{w}}'_t$ *are reverse-time Wiener processes,* $\boldsymbol{\mu}, \boldsymbol{\mu}' : \mathbb{R}^d \times [0,1] \to \mathbb{R}^d$*, and* $g : [0,1] \to \mathbb{R}_{>0}$*. Assume that* $p_1 = p'_1$*, that both SDEs admit strong solutions, and that* $\mathbb{P}' \ll \mathbb{P}$*, where* $\mathbb{P}, \mathbb{P}'$ *are the path measures induced by the SDEs on* $C([0,1], \mathbb{R}^d)$*. Then:*

$$D_{\mathrm{KL}}(p'_0 \| p_0) \leq \frac{1}{2} \int_0^1 \frac{1}{g_t^2}\, \mathbb{E}_{\mathbf{x}\sim p'_t} \left[\|\boldsymbol{\mu}'_t(\mathbf{x}) - \boldsymbol{\mu}_t(\mathbf{x})\|^2 \right]\, \mathrm{d}t\ . \tag{43}$$

Proof. By applying the chain rule of the KL divergence[S8] Theorem 2.4 at $t = 0$ and $t = 1$, we have:

$$D_{\mathrm{KL}}(\mathbb{P}' \| \mathbb{P}) = D_{\mathrm{KL}}(p'_0 \| p_0) + \mathbb{E}_{\mathbf{x}_0^* \sim p'_0} \Big[\underbrace{D_{\mathrm{KL}}(\mathbb{P}'_{\mathbf{x}_0 = \mathbf{x}_0^*} \| \mathbb{P}_{\mathbf{x}_0 = \mathbf{x}_0^*})}_{\geq 0} \Big] \tag{44}$$

$$D_{\mathrm{KL}}(\mathbb{P}' \| \mathbb{P}) = \underbrace{D_{\mathrm{KL}}(p'_1 \| p_1)}_{=0} + \mathbb{E}_{\mathbf{x}_1^* \sim p'_1} \left[D_{\mathrm{KL}}(\mathbb{P}'_{\mathbf{x}_1 = \mathbf{x}_1^*} \| \mathbb{P}_{\mathbf{x}_1 = \mathbf{x}_1^*}) \right]\ . \tag{45}$$

The subscripts on the path measures denote conditioning on the value of the process at a specific time (by disintegration of path measures). We can therefore bound $D_{\mathrm{KL}}(p'_0 \| p_0)$ by a KL divergence between path measures:

$$D_{\mathrm{KL}}(p'_0 \| p_0) \leq \mathbb{E}_{\mathbf{x}_1^* \sim p'_1} \left[D_{\mathrm{KL}}(\mathbb{P}'_{\mathbf{x}_1 = \mathbf{x}_1^*} \| \mathbb{P}_{\mathbf{x}_1 = \mathbf{x}_1^*}) \right]\ . \tag{46}$$

By Girsanov's theorem[S9],

$$D_{\mathrm{KL}}(\mathbb{P}'_{\mathbf{x}_1 = \mathbf{x}_1^*} \| \mathbb{P}_{\mathbf{x}_1 = \mathbf{x}_1^*}) = \frac{1}{2} \mathbb{E}_{\mathbb{P}'_{\mathbf{x}_1 = \mathbf{x}_1^*}} \left[\int_0^1 \frac{1}{g_t^2} \|\boldsymbol{\mu}'_t(\mathbf{x}_t) - \boldsymbol{\mu}_t(\mathbf{x}_t)\|^2\, \mathrm{d}t \right]\ . \tag{47}$$

We can now write the iterated expectation as an expectation over the unconditional path measure $\mathbb{P}'$:

$$\mathbb{E}_{\mathbf{x}_1^*\sim p_1'}\left[D_{\mathrm{KL}}(\mathbb{P}'_{\mathbf{x}_1=\mathbf{x}_1^*}\|\mathbb{P}_{\mathbf{x}_1=\mathbf{x}_1^*})\right] = \frac{1}{2}\mathbb{E}_{\mathbf{x}_1^*\sim p_1'}\left[\mathbb{E}_{\mathbb{P}'_{\mathbf{x}_1=\mathbf{x}_1^*}}\left[\int_0^1 \frac{1}{g_t^2}\|\boldsymbol{\mu}_t'(\mathbf{x}_t)-\boldsymbol{\mu}_t(\mathbf{x}_t)\|^2\,\mathrm{d}t\right]\right] \tag{48}$$

$$= \frac{1}{2}\mathbb{E}_{\mathbb{P}'}\left[\int_0^1 \frac{1}{g_t^2}\|\boldsymbol{\mu}_t'(\mathbf{x}_t)-\boldsymbol{\mu}_t(\mathbf{x}_t)\|^2\,\mathrm{d}t\right]\ . \tag{49}$$

Finally, we switch the expectation and integral (Fubini–Tonelli), and simplify the expectation over $\mathbb{P}'$ into an expectation over the time marginal p_t' since the argument of the integral only depends on t:

$$\mathbb{E}_{\mathbb{P}'}\left[\int_0^1 \frac{1}{g_t^2}\|\boldsymbol{\mu}_t'(\mathbf{x}_t)-\boldsymbol{\mu}_t(\mathbf{x}_t)\|^2\,\mathrm{d}t\right] = \int_0^1 \frac{1}{g_t^2}\ \mathbb{E}_{\mathbf{x}\sim p_t'}\left[\|\boldsymbol{\mu}_t'(\mathbf{x})-\boldsymbol{\mu}_t(\mathbf{x})\|^2\right]\,\mathrm{d}t\ , \tag{50}$$

which concludes the proof. □

In this work, we are specifically interested in the reverse-time SDEs (3) and (8) in main manuscript:

Proposition (Restatement of Proposition 2). Let p_t and p_t' be the distributions at time t induced by the reverse-time SDEs (3) and (8) starting from the same distribution p_1 at $t=1$. Assume that both SDEs admit strong solutions, and that $\mathbb{P}' \ll \mathbb{P}$, where $\mathbb{P}, \mathbb{P}'$ are the path measures induced by the SDEs on $C([0,1],\mathbb{R}^d)$. Then:

$$D_{\mathrm{KL}}(p_0'\|p_0) \leq \int_0^1 w_{\mathrm{KL}}(t)\ \frac{g(t)^4}{4}\ \mathbb{E}_{\mathbf{x}\sim p_t'}\left[\|\mathbf{h}_\vartheta(\mathbf{x},t)\|^2\right]\,\mathrm{d}t\ , \qquad w_{\mathrm{KL}}(t) := \frac{2}{g(t)^2}\ . \tag{51}$$

Proof. The result directly follows by applying Proposition 4 to the drifts of the reverse-time SDEs (3) and (8). □

B Experimental details

B.1 Coarse-grained Boltzmann Emulator model architecture and training setup

The score function in this work is based on the CPaiNN architecture introduced in[S10] with $n_h = 64$ hidden features and five message passing layers. The score is calculated in two steps - embedding and processing by CPaiNN. In the embedding step, each node is embedded using a lookup function. The pairwise distances between nodes and the diffusion time t is encoded with a positional embedding as described in[S11]. The embedded t is concatenated to the node features and the resulting vector is projected down to n_h dimensions using an MLP. Additionally, each node is assigned n_h zero-vectors serving as initial equivariant features.

The embedded graph is processed by the score model and the final equivariant features are read out as the score.

The score model was trained in a DDPM setup as described in[S10] using an exponential moving average[S12] with a decay value of 0.99, batch size of 128, and the Adam optimizer with a learning rate of 0.001, $\beta_1 = .9$, $\beta_2 = .999$.

B.2 Analysis of CLN025 MD Trajectory

To evaluate our methods, we calculate pair-wise C^α distances of the ten-residue miniprotein and project those features onto the two slowest time-lagged independent components[S13] with a lag time $\tau = 10\,\mathrm{ns}$. We then clustered the MD trajectory into $n = 128$ states using KMeans. The discretized trajectory was then used for estimating a Markov State Model (MSM)[S14–S16] using a lag time of $\tau = 10\,\mathrm{ns}$[S17]. For detailed discussions on the background and use of these methods, we refer the reader to[S14,S18–S20].

In order to identify the transition states for, both chignolin and Protein G, we computed the committor probabilities[S21], defining transition states as those with values near 0.5. If we consider a reactive process of a system on a space Ω going from a state $A \subset \Omega$ to another

state $B \subset \Omega$, s. t. $A \cap B = \emptyset$, the committor q_i describes the probability of reaching state B before A starting from i.[S22] Considering the protein folding process, A is the unfolded state and B is the folded state, $q_i = P(\text{folded first} \mid \text{starting at state i})$. Most importantly in our context, are states with committor values near 0.5, indicating an equal likelihood of folding or unfolding, which are identified as *transition states* (Fig. S1 and Fig. 4 in main manuscript). These states often represent critical bottlenecks in the folding process or in chemical reactions and are thus of significant biophysical and chemical interest.

B.2.1 Committor Probabilities and Transition States.

B.3 Observable Guidance

We evaluated our method on two systems: a synthetic one-dimensional model and the chignolin protein system. For both systems, guidance parameters were optimized using Bayesian optimization with Gaussian Processes (GPs) implemented via scikit-optimize[S23]. The scaling function took the form $\eta_t(\vartheta) = \eta_{\text{init}} \exp(-\kappa(1 - t))$, with system-specific search spaces for η_{init} and κ. All optimizations used 64 function evaluations with a convergence threshold of 1e-5, retaining the 5 best parameter sets. The scaling hyperparameter γ, which balances observable matching and minimum excess work, was consistently set to 1e-3 after hyperparameter search.

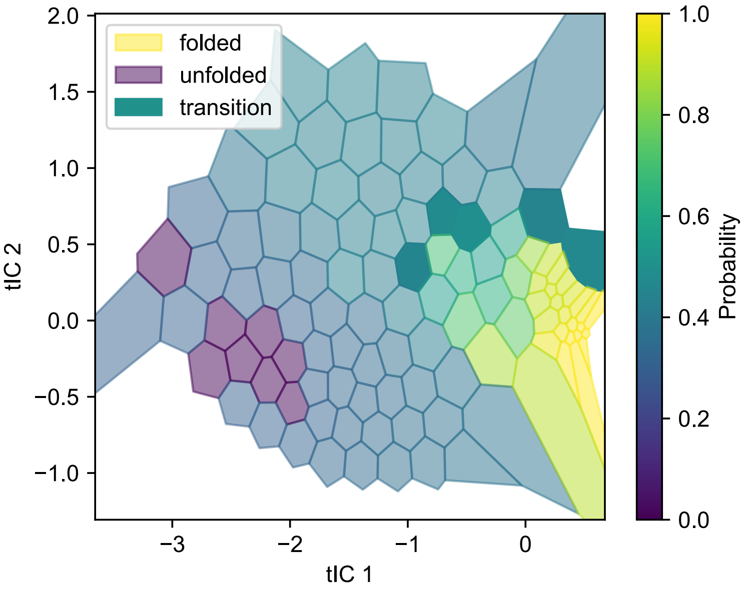

Figure S1: **Committor Probability Voronoi Diagram.** Each region is colored by its committor probability, where values near 1 correspond to folded states and values near 0 correspond to unfolded states. Regions near 0.5 represent transition states.

B.3.1 Synthetic setup and additional results

Task and data. We train a diffusion model on samples from a biased 1D quadruple-well potential[S14], allowing for direct, distribution-level validation of a system that displays multimodality and metastability yet has a numerically accessible unbiased Boltzmann distribution.

Neural Network Architecture and Training. Two multilayer perceptron (MLP) networks were trained on the Prinz potential system[S14] with $k_B = 1.38 \cdot 10^{-23}$ and $T = 300$ K: one on the unbiased potential and another incorporating a linear bias of -4. Both networks were trained for 15,000 epochs using a batch size of 256 and the Adam optimizer with a learning rate of 1e-3. The networks shared identical architectures, with input dimension corresponding to single-atom ($n_{\text{atoms}} = 1$) one-dimensional data, a time embedding dimension of 3, hidden dimension of 64, and output dimension of 1. The training process employed a linear beta scheduler with parameters $a = 0.1$ and $b = 20.0$. This scheduler controlled the noise scale during training, allowing for progressive refinement of the learned distributions.

Observable Function Parameterization. For the synthetic system, the observable function was implemented as a Gaussian Mixture Model (GMM) with four components, parameterized as shown in Table S1. The Lagrange multiplier was calculated to be -0.66 following[S6]. The parameter search space was defined as $\eta_{\text{init}} \in [1.0, 20.0]$ and $\kappa \in [1.0, 20.0]$.

Table S1: Gaussian Mixture Model Component Parameters

Component	Mean (μ)	Variance (σ^2)	Weight (w)
1	0.30	0.01	0.35
2	-0.24	0.01	0.22
3	0.69	0.01	0.27
4	-0.71	0.01	0.16

Evaluation metrics. We report (i) $\mathbb{E}_{p_{\mathcal{M}}(\mathbf{x})}[O(\mathbf{x})]$ to assess constraint satisfaction and (ii) $\mathrm{KL}(p_{\text{GT}} \parallel p_{\mathcal{M}})$ to assess distributional fidelity relative to the ground truth (GT). We compare a biased reference model, the guided model, and GT.

Main result. As shown in Fig. S2, guidance corrects the biased density toward GT. Quantitatively (Table S2), KL is reduced by $\sim 10\times$ (from 0.13 to 0.019$\pm$0.002) and the observable expectation moves from -13.6 to 11.95$\pm$0.22, closely matching GT 12.01.

Ablation on MEW regularization. We compare training with ($\gamma > 0$) and without ($\gamma = 0$) MEW. Both variants match the observable expectation, but without MEW we observe mode collapse with mass concentrated in a narrow region and elevated KL, indicating poor distributional fidelity. MEW preserves the broader reference shape and stabilizes training (visuals in Fig. S10; summary metrics in Table S3).

Conclusion. This synthetic experiment demonstrates that observable guidance recovers the correct distribution using only expectation values, while MEW regularization prevents degenerate solutions and stabilizes training.

B.3.2 cgBE: Chignolin

For the chignolin protein system, we defined the observable function using the interatomic distance between the first and last C^α atoms (C_1^α and C_{10}^α). The folding free energy was calculated as:

$$\Delta G = -k_B T \log\left(\frac{p_f}{1-p_f}\right) \tag{52}$$

where p_f represents the fraction of folded samples, defined using a distance cutoff of 7.5 Å. The Lagrange multiplier was determined to be -0.5[S6]. The parameter search space was set to $\eta_{\text{init}} \in [10^{-2}, 1.0]$ and $\kappa \in [1.0, 10.0]$. The optimization process used 256 samples per epoch, with final evaluation conducted on 256×256 samples to ensure robust statistical assessment.

B.3.3 BioEmu: Homeodomain

For the homeodomain experiments, we used experimental ^3J-couplings:

$$^3J_{\mathrm{HN-HA}}(\phi) = A\,\cos^2\big(\phi - \phi_0\big) + B\,\cos\big(\phi - \phi_0\big) + C, \qquad \phi_0 = 60^\circ \tag{53}$$

using the standard parameterization of Vuister & Bax: $A = 6.98$, $B = -1.38$, $C = 1.72$. Due to high observable covariance, we selected a subset (10/43) of the most informative observables for guidance. This was done via covariance analysis, which identifies redundant measurements that provide overlapping structural information and reveals the effective dimensionality of the conformational space sampled by the observables (see Fig. S6). To identify these observables, we performed PCA on the covariance matrix and identified observables that contribute significantly to multiple principal components (threshold: $|\text{loading}| > 0.25$, see Fig. S7). Determining the Lagrange multipliers was done using,[S24] and the effective sample size was found to be 0.255. BioEmu's internal representation consists of a position matrix $\mathbf{r}$ and a rotation matrix $\mathbf{Q}$.[S25] Our augmenter module operates directly on the $(\mathbf{r}, \mathbf{Q})$ tuple representation of positions and residue orientations. From these coarse-grained coordinates, we reconstruct the backbone geometry, which allows us to compute experimental observables such as the dihedral angle ϕ required for $^3J_{\mathrm{HN-HA}}$-couplings. The augmenter then evaluates the weighted experimental loss and provides its gradients with respect to both $\mathbf{r}$ and $\mathbf{Q}$. For positions, this yields standard Euclidean forces. For orientations, gradients are first mapped to the Lie algebra $\mathfrak{so}(3)$, ensuring that all updates remain consistent with the $SO(3)$ manifold structure. During denoising, the augmenter is evaluated not on the noisy state $(\mathbf{r}_t, \mathbf{Q}_t)$ but on Tweedie posterior mean estimates of the clean structure. For positions, the Tweedie relation

$$\hat{\mathbf{r}}_0 \;=\; \frac{\mathbf{r}_t + \sigma_t^2\, s_t^{(r)}}{\alpha_t} \tag{54}$$

links the network's position score $s_t^{(r)}$ to an estimate of the clean coordinates $\hat{\mathbf{r}}_0$. For orientations, we apply an analogous manifold-aware update in $SO(3)$,

$$\hat{\mathbf{Q}}_0 \;=\; \mathbf{Q}_t \exp\Big(\widehat{\Omega}\Big(\tfrac{\sigma_t^2}{\alpha_t}\,\omega_t\Big)\Big)\,, \tag{55}$$

where ω_t denotes the predicted rotational score in axis–angle representation and $\widehat{\Omega}(\cdot)$ maps it to a skew-symmetric matrix. This update guarantees that $\hat{\mathbf{Q}}_0 \in SO(3)$ without requiring an ambient projection. In this way, observable guidance enters the diffusion model consistently for both positions and orientations, while preserving Euclidean and manifold constraints. For training, we used 3,500 samples to evaluate the observable expectations using a batch size of 700. Evaluation was done using 4,900 samples. γ was set to 1e-3 and the parameter search space was set to $\eta_{\text{init}} \in [10^{-2}, 1.0]$ and $\kappa \in [3.0, 10.0]$.

B.3.4 Compute Resources and Runtime Details

Observable guidance experiments were conducted using HPC compute infrastructure equipped with NVIDIA A100 GPUs (80GB memory). Training and evaluation scripts were run on single-GPU nodes.

For the synthetic system (Section S2 and Table S2), each experiment took approximately 4 min to run, consuming 6 GB GPU memory. The chignolin experiments (e.g., Figure S13) required up to 30 min of compute time per run, and 30 GB of GPU memory due to the larger input size and batch requirements. Ablation studies (Figure S10) were conducted with the same hardware and each variant was run across 50 (synthetic case) and 10 (chignolin case) seeds, requiring 1–3 hours per configura-

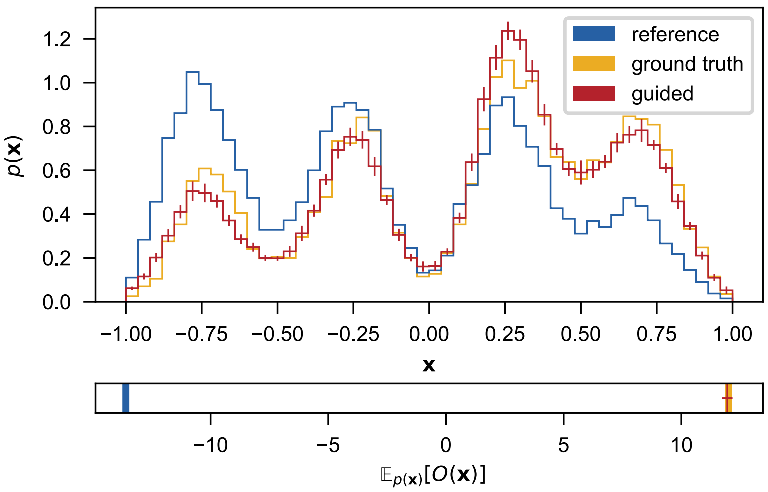

Figure S2: **Comparison of Probability Distributions Before and After Observable Guidance for a 1D Energy Potential.** The top plot shows the probability distributions for three models: the biased reference model (blue), the ground truth model (yellow), and the guided model (red). Guidance helps to align the reference model with that of the ground truth model using only the expectation of an observable function (bottom) while minimizing excess work.

tion. In total, the reported experiments required approximately 10 GPU hours. Preliminary runs and failed hyperparameter sweeps amounted to an estimated additional 100 GPU hours, not included in the main results. All experiments were executed in a reproducible virtual environment with pinned dependencies (provided in the supplemental code release).

B.4 Path Guidance

Similar to observable guidance, path and loss guidance were evaluated on two systems: a synthetic two-dimensional setup and the chignolin mini-protein. We experimented with various functional forms for the guiding strength and time-dependent bandwidth and found that sigmoid-like step functions performed well across both tasks:

$$\eta_t(\vartheta) = \vartheta_{init}\,(1 - \sigma(\vartheta_g(t - \vartheta_s))) \tag{56}$$

$$h_t(\varphi) = \varphi_{init} + \sigma(\varphi_g(t - \varphi_s)) \tag{57}$$

To optimize the parameter sets ϑ and φ we applied Bayesian optimization with Gaussian Processes (GPs), using the `scikit-optimize` library. We employed the `gp_hedge` acquisition function, which dynamically combines strategies such as Expected Improvement (EI), Probability of Improvement (PI), and Lower Confidence Bound (LCB) based on their empirical performance. After initial exploration, we restricted the search space to a sensible domain to improve optimization efficiency and support a broader sweep of experimental configurations.

B.4.1 Synthetic System

We evaluated our method on a simple three-moon example, where the two-dimensional dataset consists of three noisy half-moon arcs generated by sampling from shifted semicircles with optional convexity and added Gaussian noise (see Fig. S11). While two of the arcs are well-represented in the training data, only 2.5% of the samples belong to the third arc, creating a challenging low-data region. We adopt the Conditional Flow Matching (CFM) framework[S26],

from which the score function can be derived for augmentation. To approximate the resulting vector field, we train a four-layer MLP on 10,000 samples for 3,000 steps using a learning rate of 10^{-4} and a batch size of 256. Training hyperparameters were selected via a small grid search on an NVIDIA A100 GPU. For optimizing the guidance schedules in Eqs. (56) and (57), we run 25 Bayesian optimization steps. To classify whether a sample falls within the target moon, we train a two-layer MLP classifier using a learning rate of 10^{-3}, 1,000 training steps, and a batch size of 256. For path and loss guidance, we evaluated γ values between 0 and 1, finding 0.03 working best for path guidance and 0.1 for loss guidance. For sampling we use 20 guiding points generating 1000 samples in one batch.

B.4.2 Chignolin System

The Boltzmann Emulator used for sampling the chignolin system is described in Appendix B.1. Since loss guidance could not be reliably optimized via Bayesian optimization, we performed an extensive grid search over hyperparameters, including various functional forms for the schedules in Eqs. (56) and (57). This grid search was run for 24 hours on a single NVIDIA H100 GPU and served primarily to investigate the failure modes of loss guidance. The corresponding results are shown in Fig. S13B. To improve stability, we explored gradient clipping and found it essential for loss guidance. For MEW-guided optimization, we focused exclusively on path guidance. We tested γ values between 0 and 1 and found values $\gamma \leq 0.5$ to be effective. Each run consisted of 50 Bayesian optimization steps, with one function evaluation taking approximately 2.5 minutes. As a result, a full optimization run for a fixed γ required about two hours on a single NVIDIA H100 GPU (80GB). After each iteration, we computed committor probabilities of the sampled protein conformations using the method described in Appendix B.2 to estimate the proportion of transition-state configurations. For each of the 50 guiding points available, we generated 10 samples, leading to a sample batch size of 500.

B.4.3 BioEmu: Protein G

BioEmu models both backbone geometry and local frames, diffusing translations $r \in \mathbb{R}^3$ and orientations $Q \in SO(3)$. While we reuse the Euclidean path-guidance construction for translations, we extend it to orientations by defining a KDE-style guidance term in the rotation tangent space $\mathfrak{so}(3)$.

At diffusion time t, let $Q_t \in SO(3)$ denote a residue's current orientation and let $\{Q_t^{g,i}\}_{i=1}^M$ be the corresponding guiding orientations (i.e., the orientation components of X_t^g). We form *right-trivialized* relative rotations

$$\Delta_t^i \;=\; \mathrm{Log}\big(Q_t^\top Q_t^{g,i}\big) \;\in\; \mathfrak{so}(3), \tag{58}$$

(and identify Δ_t^i with its axis–angle vector in $\mathbb{R}^3$ when taking norms). Using a Gaussian kernel with time-dependent bandwidth $h_t^{(Q)}(\varphi)$,

$$k_t^i \;\propto\; \exp\!\left(-\frac{\|\Delta_t^i\|^2}{2\left(h_t^{(Q)}(\varphi)\right)^2}\right), \qquad w_t^i \;=\; \frac{k_t^i}{\sum_j k_t^j}, \tag{59}$$

the corresponding rotational perturbation is then

$$\mathbf{h}_{\vartheta,\varphi}(Q_t, t) \;=\; \eta_t^{(Q)}(\vartheta) \sum_i w_t^i \, \frac{\Delta_t^i}{\left(h_t^{(Q)}(\varphi)\right)^2}, \tag{60}$$

which we add to BioEmu's predicted rotational score prior to the integration step. Separate time-dependent schedules are used for positional vs. rotational strength and bandwidth, both parameterized in the same functional forms as Eqs. (56) and (57), but with independent parameter sets for translations and rotations. The experimental setup is the same as in Appendix B.4.2, but due to the increased number of parameters, we use 250 Bayesian optimisation steps. Therefore, to run optimisation for a fixed value of γ, we require about 4 hours on a single NVIDIA H100 GPU. For sampling, we use BioEmu's default configuration of 100 sampling steps using the Heun solver.

C Results: Observable Guidance

All error bars for observable guidance were calculated as the standard deviation between n runs ($n = 50$ for the 1D energy potential experiments and $n = 10$ for the chignolin experiments.

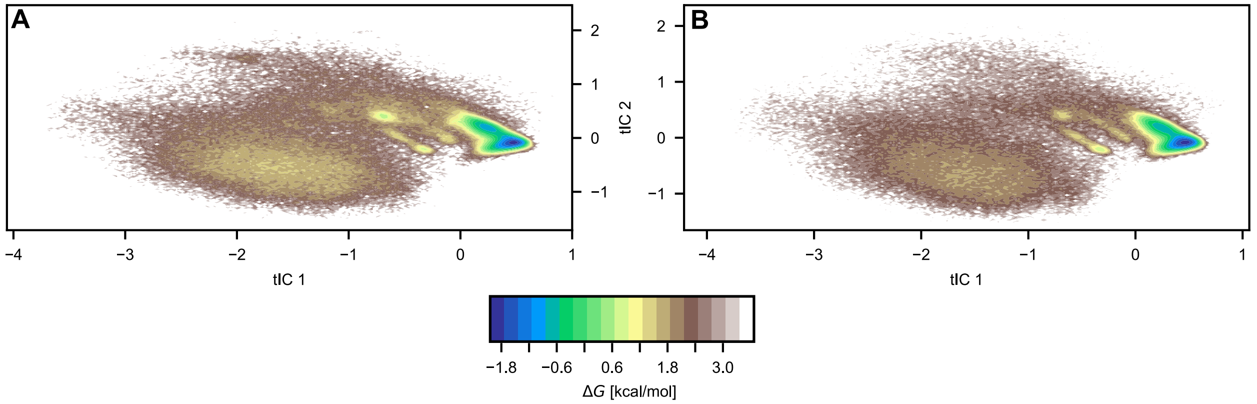

Figure S3: **tICA Projection of Original and Observable-Guided Model.** State space distribution projected onto the first and second tICs for the original (A) and guided (B) BG. The plots are colored by their respective energies.

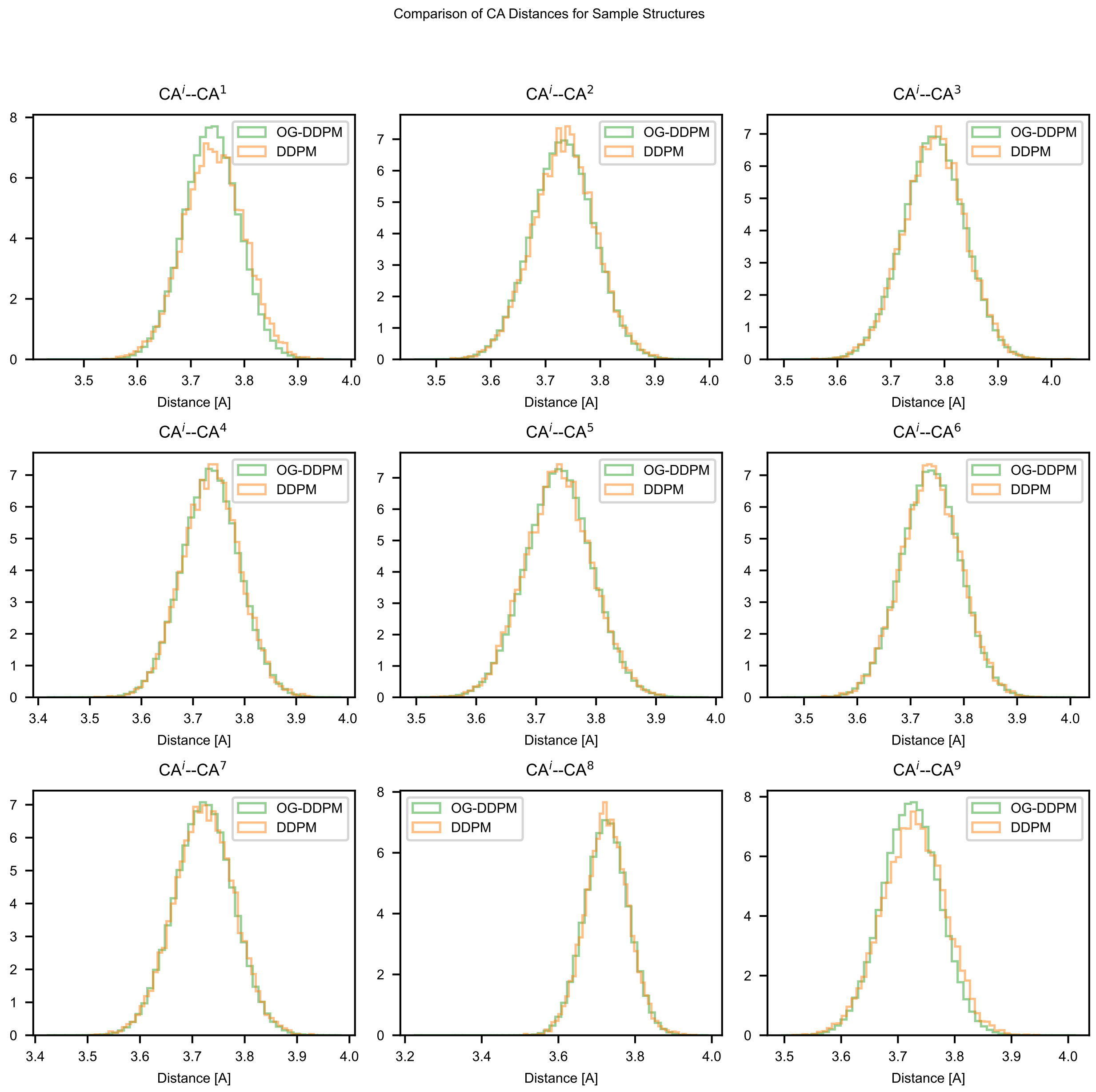

Figure S4: **Comparison of sequential C^α–C^α distances between the observable-guided diffusion model (OG-DDPM, green) and the original diffusion model (DDPM, orange).** The plots show the distance distributions for all adjacent C^α pairs (0–2 through 8–9 using zero indexing) in the protein backbone, showing that the guided model maintains proper protein geometry while achieving the desired constraints.

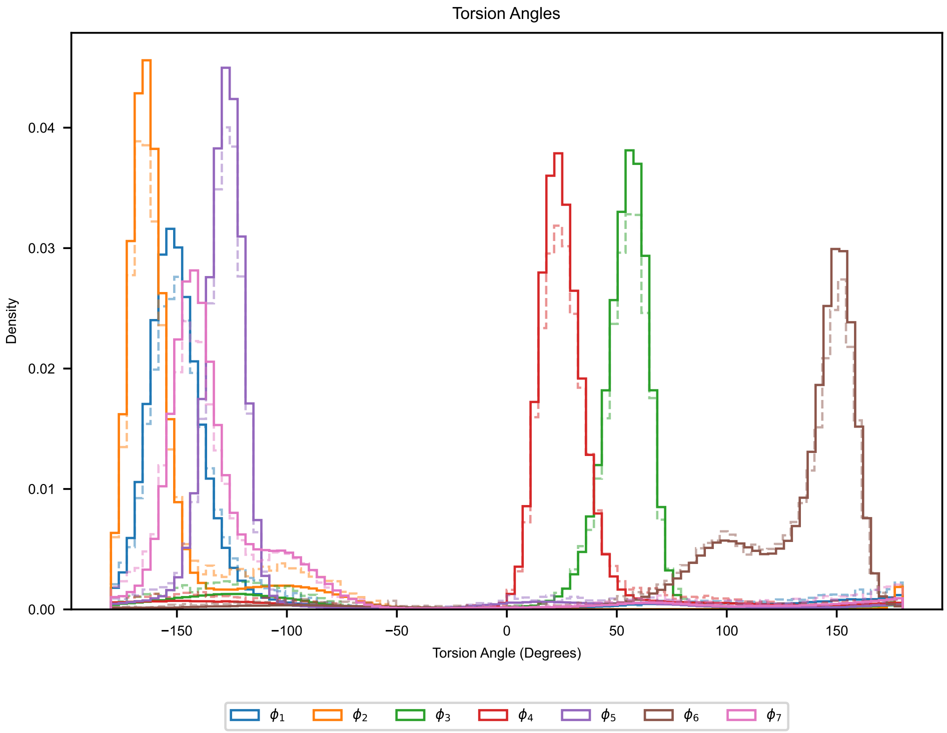

Figure S5: **Distribution of backbone torsion angles (ϕ_1 through ϕ_7) comparing the MD simulation (solid lines) with the observable-guided model (dashed lines).** The close agreement between the distributions indicates that the guided model preserves the native conformational preferences of the protein while satisfying the experimental constraints. Each torsion angle is shown in a different color. The differences between the two densities stems from the guidance procedure. Importantly, the torsion angles themselves remain the same.

C.1 Ablation Studies

D Results: Path Guidance

D.1 Synthetic System

Before applying our method to the Boltzmann Generator on the chignolin system, we first evaluated it on a simple three-moon example (Fig. S11; see Appendix B.4.1 for implementation details). This setup offers a useful testbed, as the low-data region is connected to a high-density area while remaining well-separated from the other half-moon. The objective of

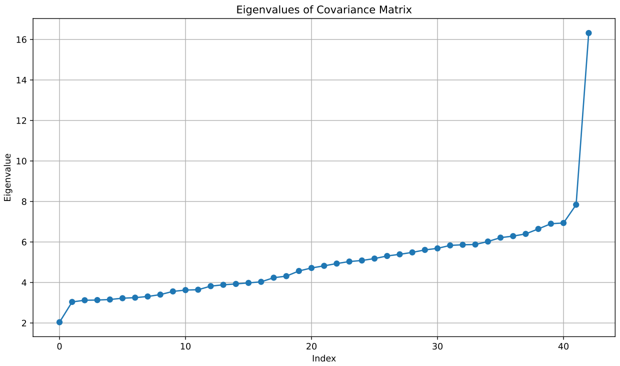

Figure S6: **Eigenspectrum of Observable Covariance Matrix.** The spectrum shows a high correlation between the observables, indicating that most carry redundant information.

Table S2: Metrics for $O(\mathbf{x})$ and KL divergence across models (synthetic).

Model $\mathcal{M}$	$\mathbb{E}_{p_{\mathcal{M}}(\mathbf{x})}[O(\mathbf{x})]$	$\mathrm{KL}(p_{\mathrm{GT}} \| p_{\mathcal{M}})$
Ground Truth	12.01	—
Reference	-13.6	0.13
Guided	11.95 ± 0.22	0.019 ± 0.002

guidance in this case is to enable transitions into the low-density region without deviating off the underlying data manifold connecting the moons.

We observe that with ODE sampling, points frequently fall off the manifold, and only careful tuning of the guiding strength minimizes this issue. In contrast, SDE guiding is more robust, as noise helps correct guidance errors. Overall, after minimal optimization of η_t and h_t, both Path Guidance and Loss Guidance perform well on this toy example. However, in both methods, careful calibration of the guiding strength at low t is essential, as errors at this stage cannot be corrected later. Hence, we found the sigmoid function to be effective in these scenarios, as it naturally converges to 0 for $t \to 1$. In contrast to the Chignolin experiment, we find that loss guidance performs equally well in this synthetic setting, likely due to the

Table S3: Metrics for $O(\mathbf{x})$ and KL divergence with and without MEW regularization.

Model $\mathcal{M}$	$\mathbb{E}_{p_{\mathcal{M}}(\mathbf{x})}[O(\mathbf{x})]$	$\mathrm{KL}[p_{\mathrm{GT}}(\mathbf{x}) \| p_{\mathcal{M}}(\mathbf{x})]$
w/o MEW	0.131	0.754 ± 1.533
w/ MEW	0.131	0.029 ± 0.007

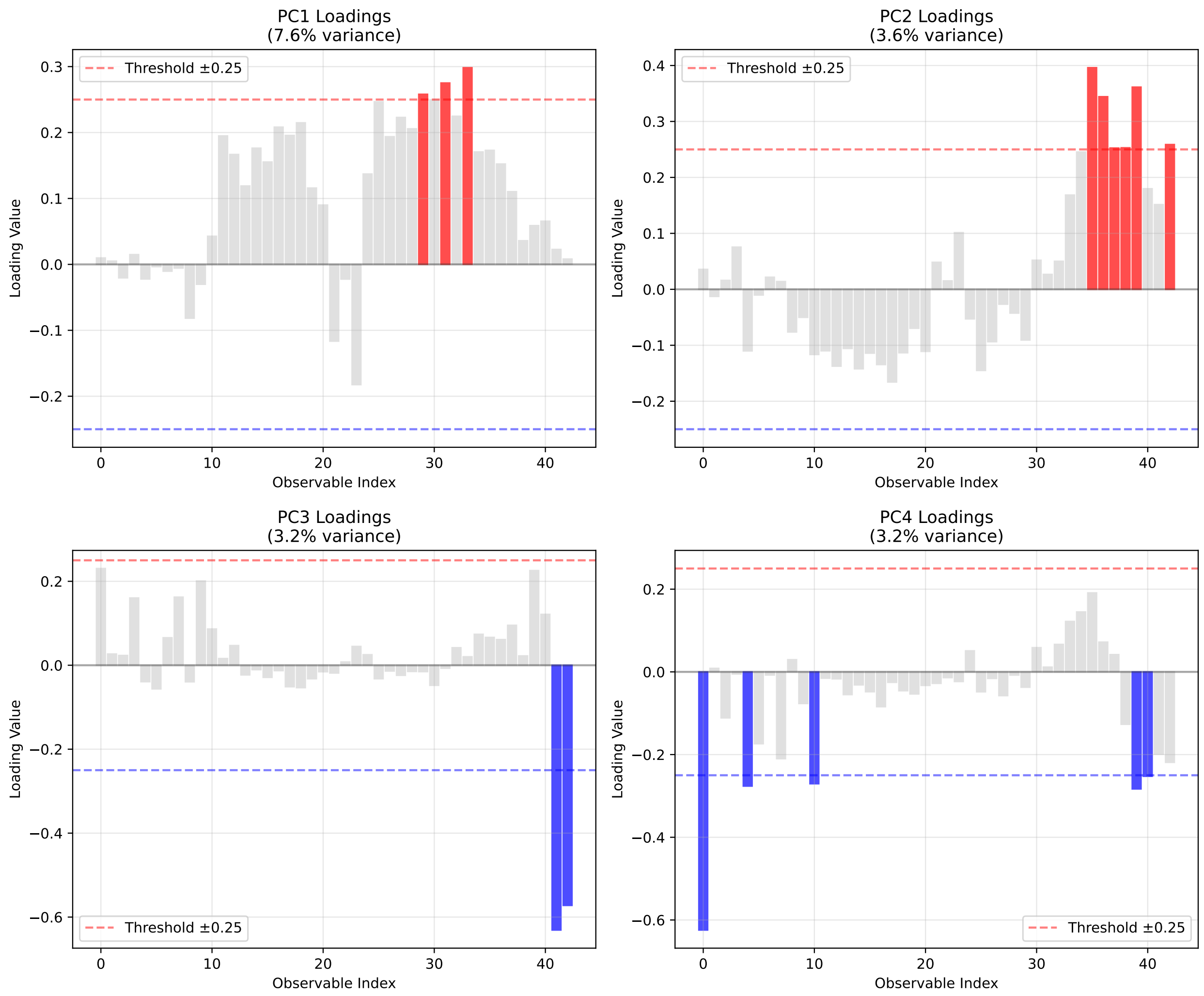

Figure S7: **Principal Components Loading Analysis for Observable Covariance.** The four biggest PCs are shown with a cut-off at 25 % to separate significant contributions to the observables.

simplicity of the data distribution, where the (KDE) in data space sufficiently captures the underlying probability distribution. We also investigate the effect of MEW regularization and observe that omitting the regularization reduces the diversity of the generated samples. Without MEW, the samples tend to be overly guided towards the guiding points on most probable regions, failing to capture the full variance of the underlying distribution Fig. S12.

D.2 Ablation Studies on Loss guidance

Since reliable sampling with loss guidance could not be achieved, we conducted a more thorough investigation to enable a fair comparison. Instead of relying on Bayesian optimization, we performed an extensive grid search over the guiding parameters (see Appendix B.4.2 for details), with particular focus on smaller guiding strengths to mitigate the effects of unstable or misaligned gradients. Compared to path guidance, the grid search results show substantially lower guiding success, with a maximum transition-state sampling rate of only 0.15%. While this does represent an improvement over unguided sampling (1%), most configurations with non-negligible guidance success resulted in degenerate samples (Fig. S13B). Our analysis suggests that while loss guidance can partially align the model with the target angle distribution, it struggles to follow the desired sampling trajectory throughout the generative process. As a result, strong corrections near the data distribution are required, increasing the risk of sample degeneration (Fig. S13A).

D.3 Baseline Experiments

In this section, we describe the other two baselines, mentioned in Appendix D.2, which do not augment the vector field. Instead, they utilize the latent representations of the guiding points $\mathcal{X}_t^g$ to initialize the sampling process for generating new points with similar latent characteristics. While these methods are appealing in their simplicity, they lack direct control over the sampling process itself.

Latent-KDE (L-KDE). We can fit a KDE in the latent space on $\mathcal{X}_1^g$, sample from it, and integrate the probability flow ODE backwards in time. Fitting the KDE at the prior can be advantageous because the Euclidean distance, on which most kernels are based, is better suited for Gaussian-distributed data compared to its use in data space. We refer to this method as Latent-KDE (L-KDE).

Stochastic-Reverse (SR). Alternatively, we can select a specific time step t such that the desired properties are preserved and initialize the backward SDE (Eq. 1 in main manuscript) with latents from $\mathcal{X}_t^g$. The stochasticity of the SDE will ensure we generate new divers samples with $\mathbf{x}' \in A$.

We conduct sampling experiments using the aforementioned baseline methods to verify whether the results align with our intuition. Specifically, for the L-KDE baseline, we evaluate a Gaussian kernel with noise levels (standard deviations) of $\{0.01, 0.05, 0.1\}$. For the SR baseline, we consider intermediate times $\{0.1, 0.5, 0.9\}$. For simplicity, we only examine the scenario where there is a single guiding point (i.e., $\mathcal{X}_1^g$ and $\mathcal{X}_t^g$ are singleton sets). Each experiment is repeated with five different seeds.

In the following figures (Figures Fig. S14 – Fig. S19), we provide various metrics, histograms, and energy surfaces that summarize the trends observed in these baseline guidance scenarios. Overall, the results strongly suggest that guidance biases the sampling procedure toward the reference guiding points, which aligns with our intuition.

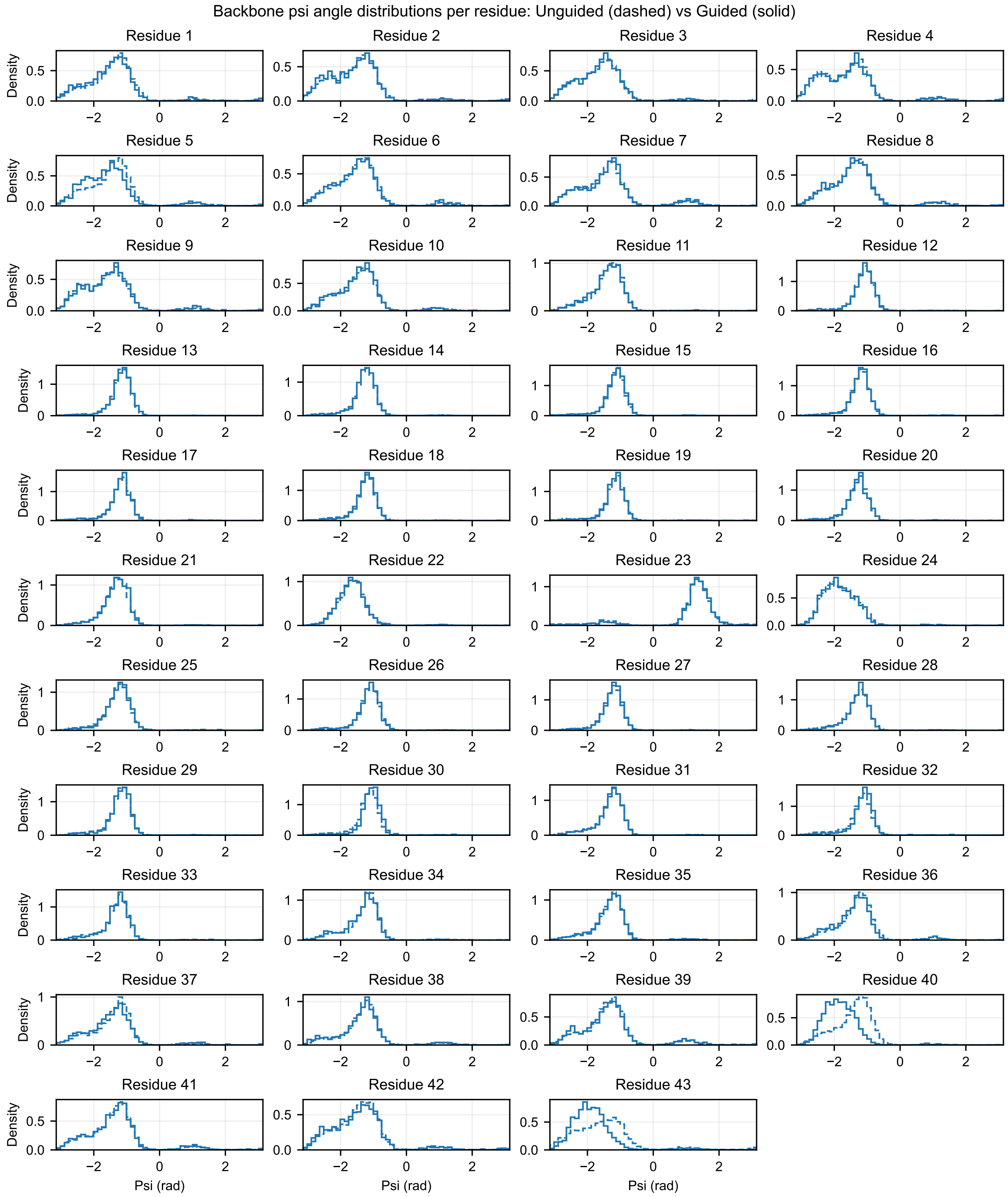

Figure S8: ϕ **backbone distribution before and after guidance.** The close agreement between the distributions indicates that the guided model preserves the native conformational preferences of the protein while satisfying the experimental constraints. The differences between the two densities stems from the guidance procedure. Importantly, the torsion angles themselves remain the same.

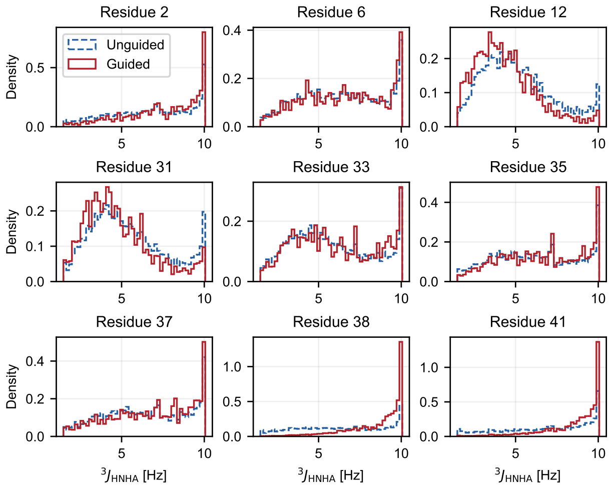

Figure S9: **Distribution of Observables Used in Guidance.** The plots show the distribution of the observables as a function of state space. Blue indicates predictions from the original BioEmu model and red are the MEW-regularized predictions.

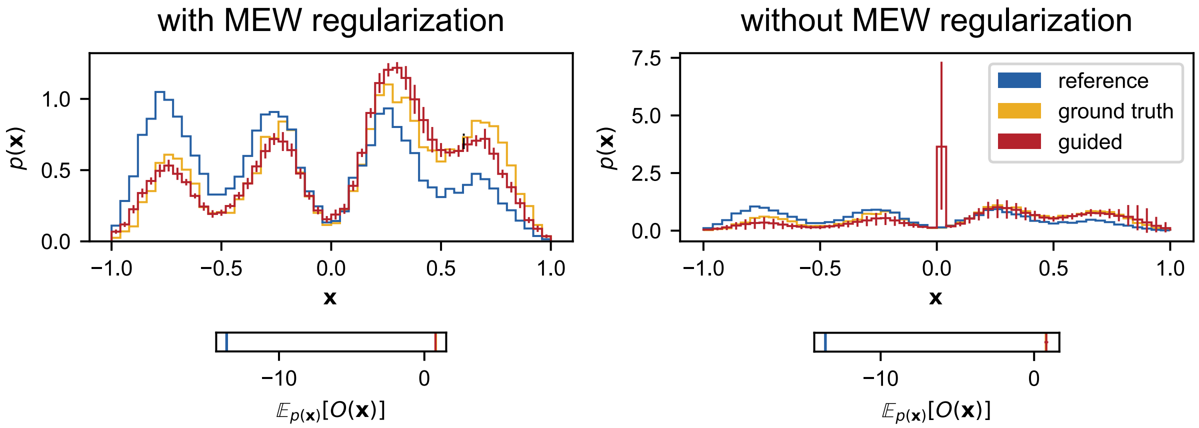

Figure S10: **Ablation study on MEW regularization in the 1D four-well potential.** Left: With MEW regularization, the guided distribution (red) closely matches both the reference (blue) and ground truth (yellow) distributions. Right: without regularization, guidance leads to mode collapse and overconcentration, resulting in low observable prediction error but poor distributional fidelity. Insets show the expected observable values $\mathbb{E}_{p(\mathbf{x})}[O(\mathbf{x})]$.

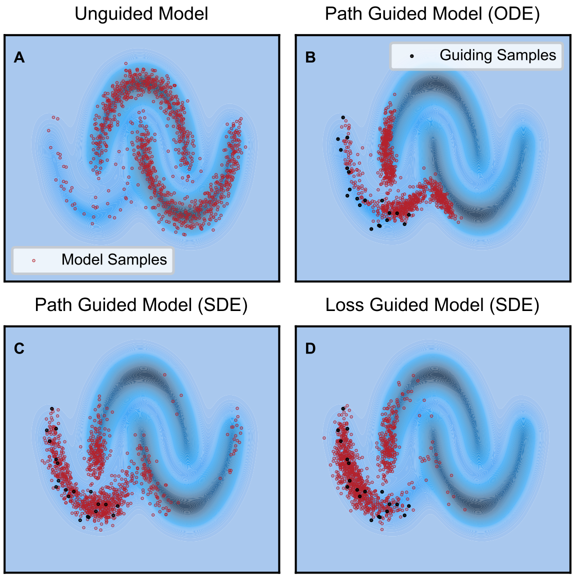

Figure S11: **Sampling the synthetic system.** Comparison of unguided, path-guided, and loss-guided models using both SDE and ODE samplers.

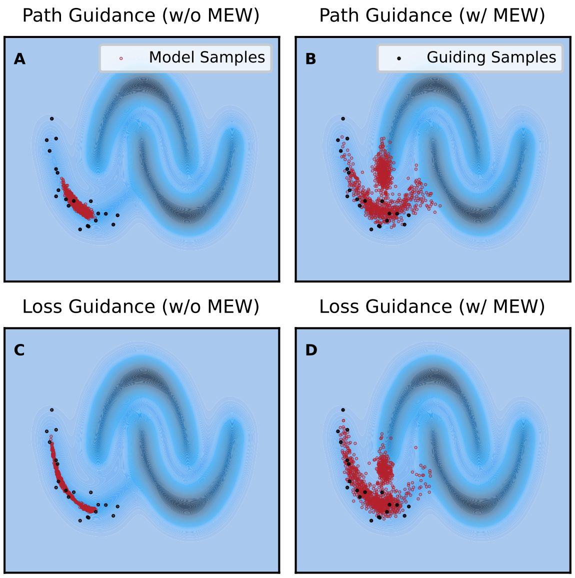

Figure S12: **Guidance with and without MEW regularization**. MEW guidance ensures that we do not collapse onto the guiding points.

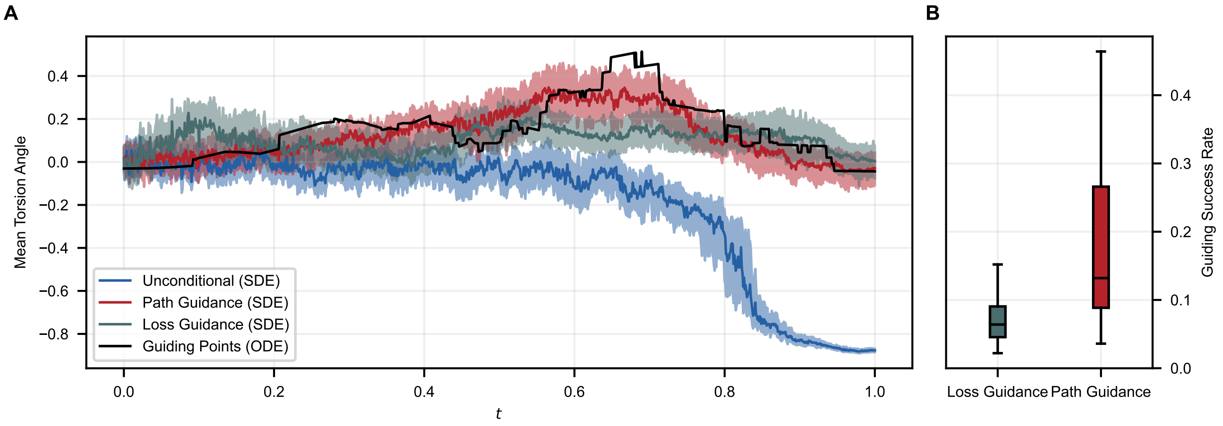

Figure S13: **Path Guidance vs. Loss-Guidance for sampling Transition States**. (A) Evolution of the mean torsion angle (which determines the state of the protein) during the diffusion process. (B) Success rates across different parameter settings.

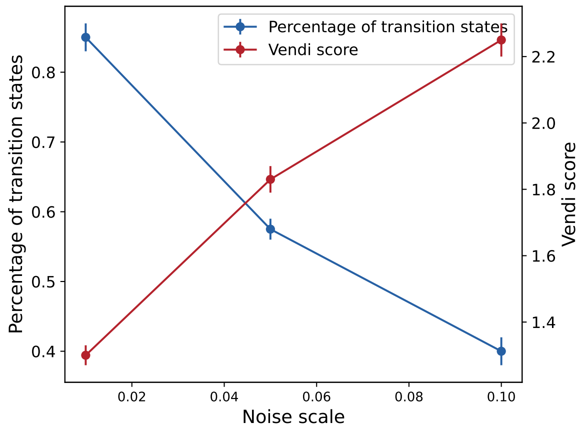

Figure S14: **Trade-off between sample variance and guidance success rate (L-KDE).** As the KDE noise scale increases for the L-KDE baseline, the percentage of transition states among the generated samples decreases (blue), while the vendi score among the generated states increases (red).

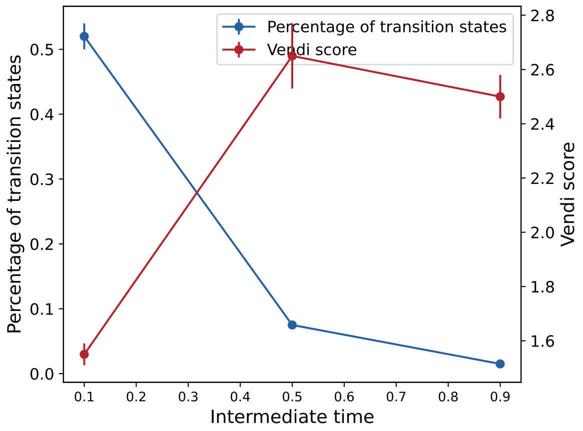

Figure S15: **Trade-off between sample variance and guidance success rate (SR).** As the selected time step t increases for the SR baseline, the percentage of transition states among the generated samples decreases (blue), while the vendi score among the generated states tends to increase (red).

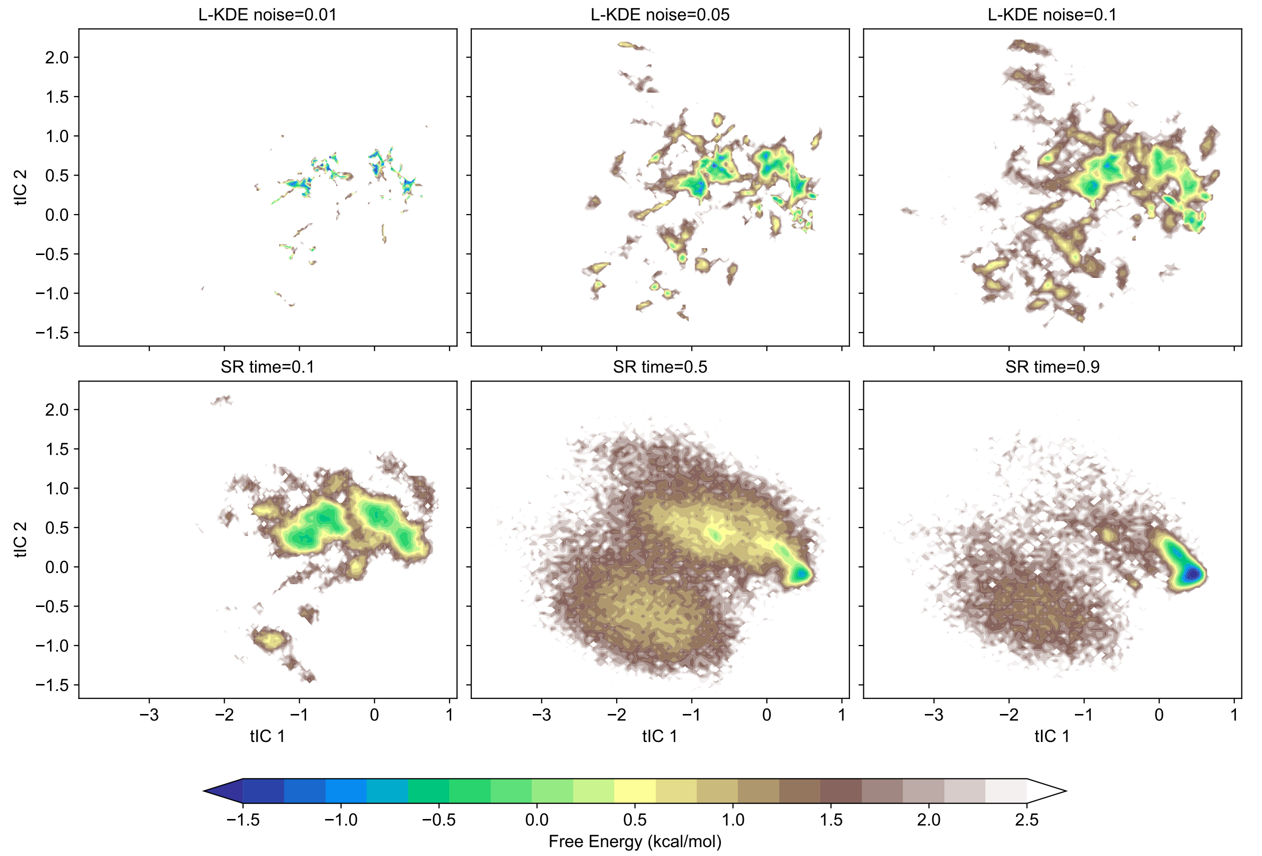

Figure S16: **Energy surface plots.** First row: L-KDE baseline for various levels of noise scale. The smaller the perturbation, the more concentrated the samples around the transition states region. Second row: SR baseline for various values of intermediate time. The smaller the stochasticity level, the more concentrated the samples around the transition states region. Compare with Fig. S1 and Fig.1 A and B in main manuscript.

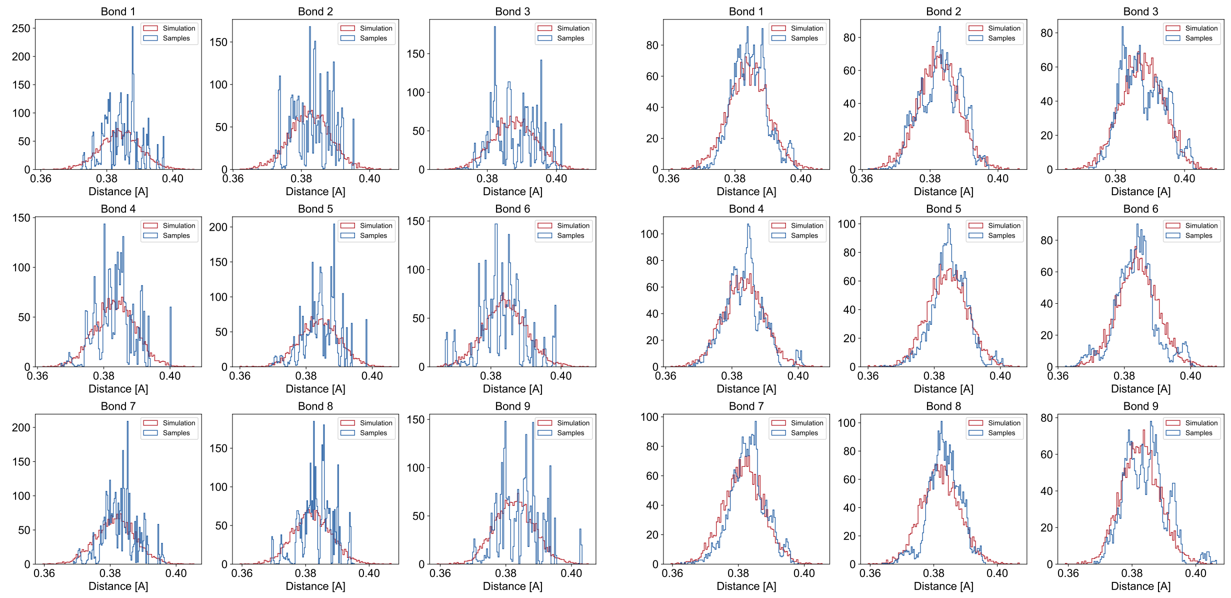

(a) L-KDE noise=0.01

(b) L-KDE noise=0.1

Figure S17: **Comparison of bond distance distributions for L-KDE and the reference.** The L-KDE baseline (blue) is superposed on the corresponding histogram of the unconditional (red) distribution (the CLN025 MD simulation). We see that for small perturbations, the generated samples seem to conform to particular details of the guiding samples. As the noise increases, the guidance impact diminishes. This is quantified in a more principled way in Fig. S19.

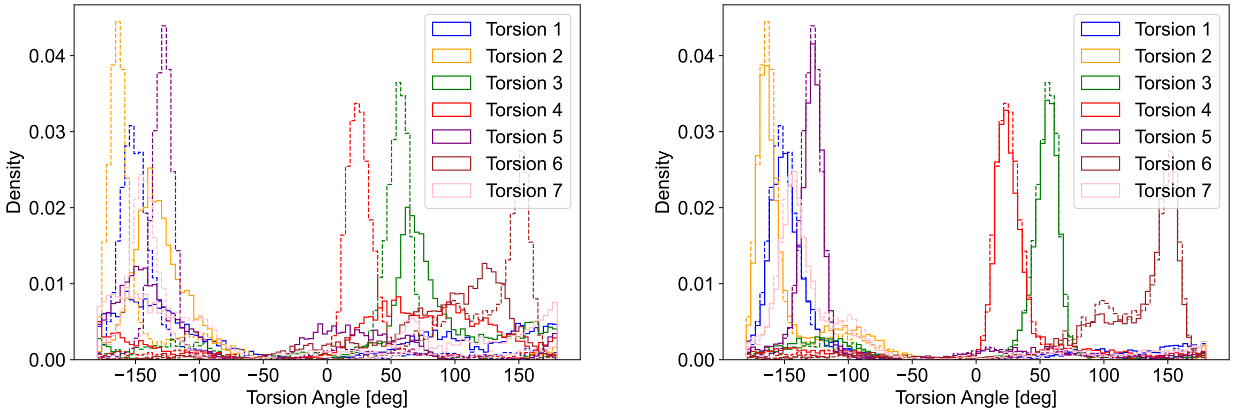

(a) SR intermediate time=0.1

(b) SR intermediate time=0.9

Figure S18: **Torsion angle histograms for the SR baseline at different noise levels.** Solid lines show SR samples at $t = 0.1$ (Left) and $t = 0.9$ (Right), superimposed on the corresponding unconditional distributions (dashed lines). At high stochasticity ($t = 0.9$), the torsion angle distribution becomes nearly indistinguishable from the unconditional one.

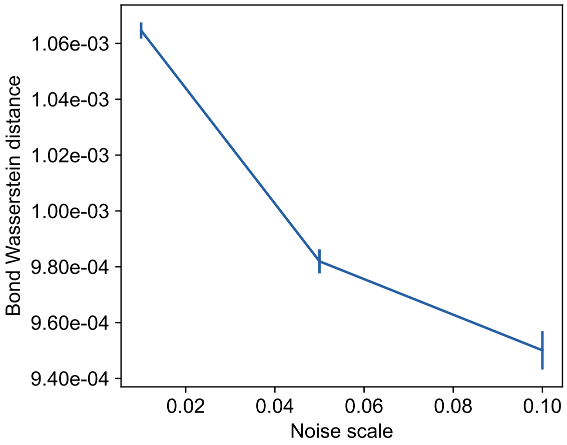

(a) Wasserstein distance vs noise scale for L-KDE baseline

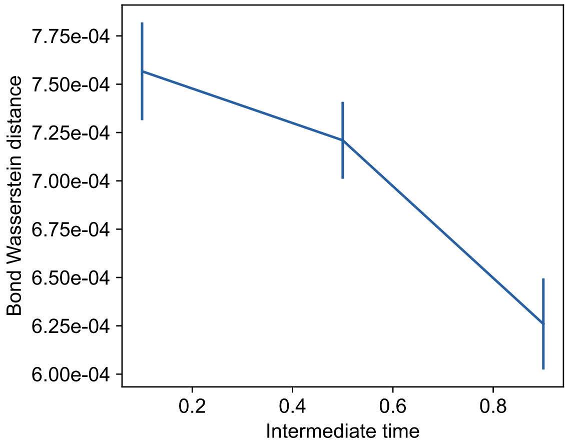

(b) Wasserstein distance vs intermediate time for SR baseline

Figure S19: **Wasserstein distance between baselines and reference bond distance distributions.** We measure the distance between the bond distance distributions of baseline methods and the CLN025 MD simulation (see Fig. S17). As the stochasticity level increases for both baselines, the generated distributions converge toward the unconditional reference, indicating a reduced influence of the guidance signal.

References

(S1) Jaynes, E. T. Information theory and statistical mechanics. *Physical review* **1957**, *106*, 620.

(S2) Hummer, G.; Köfinger, J. Bayesian ensemble refinement by replica simulations and reweighting. *The Journal of chemical physics* **2015**, *143*.

(S3) Boomsma, W.; Ferkinghoff-Borg, J.; Lindorff-Larsen, K. Combining experiments and simulations using the maximum entropy principle. *PLoS computational biology* **2014**, *10*, e1003406.

(S4) Olsson, S.; Strotz, D.; Vögeli, B.; Riek, R.; Cavalli, A. The dynamic basis for signal propagation in human Pin1-WW. *Structure* **2016**, *24*, 1464–1475.

(S5) Olsson, S.; Wu, H.; Paul, F.; Clementi, C.; Noé, F. Combining experimental and simulation data of molecular processes via augmented Markov models. *Proceedings of the National Academy of Sciences of the United States of America* **2017**, *114*, 8265–8270.

(S6) Bottaro, S.; Bengtsen, T.; Lindorff-Larsen, K. Integrating molecular simulation and experimental data: a Bayesian/maximum entropy reweighting approach. *Structural bioinformatics: methods and protocols* **2020**, 219–240.

(S7) Bressan, A.; Piccoli, B. *Introduction to the mathematical theory of control*; American institute of mathematical sciences Springfield, 2007; Vol. 1.

(S8) Léonard, C. Some properties of path measures. *Séminaire de Probabilités XLVI* **2014**, 207–230.

(S9) Øksendal, B. *Stochastic Differential Equations: An Introduction with Applications*, 6th ed.; Springer, 2003.

(S10) Schreiner, M.; Winther, O.; Olsson, S. Implicit Transfer Operator Learning: Multiple Time-Resolution Models for Molecular Dynamics. Thirty-seventh Conference on Neural Information Processing Systems. 2023.

(S11) Vaswani, A.; Shazeer, N.; Parmar, N.; Uszkoreit, J.; Jones, L.; Gomez, A. N.; Kaiser, Ł.; Polosukhin, I. Attention is all you need. *Advances in neural information processing systems* **2017**, *30*.

(S12) Tarvainen, A.; Valpola, H. Mean teachers are better role models: Weight-averaged consistency targets improve semi-supervised deep learning results. *Advances in neural information processing systems* **2017**, *30*.

(S13) Pérez-Hernández, G.; Paul, F.; Giorgino, T.; De Fabritiis, G.; Noé, F. Identification of slow molecular order parameters for Markov model construction. *The Journal of chemical physics* **2013**, *139*.

(S14) Prinz, J.-H.; Wu, H.; Sarich, M.; Keller, B.; Senne, M.; Held, M.; Chodera, J. D.; Schütte, C.; Noé, F. Markov models of molecular kinetics: Generation and validation. *The Journal of chemical physics* **2011**, *134*.

(S15) Bowman, G. R.; Pande, V. S.; Noe, F. *An Introduction to Markov State Models and Their Application to Long Timescale Molecular Simulation (Advances in Experimental Medicine and Biology*, volume 797 ed.; Springer Science+Business Media Dordrecht, 2014.

(S16) Kolloff, C.; Olsson, S. Machine Learning in Molecular Dynamics Simulations of Biomolecular Systems. *Comprehensive Computational Chemistry* **2024**, *3*, 475–492.

(S17) Hoffmann, M.; Scherer, M.; Hempel, T.; Mardt, A.; de Silva, B.; Husic, B. E.; Klus, S.; Wu, H.; Kutz, N.; Brunton, S. L.; Noé, F. Deeptime: a Python library for machine learning dynamical models from time series data. *ArXiv* **2021**, *3*, 1–32.

(S18) Bowman, G. R.; Beauchamp, K. A.; Boxer, G.; Pande, V. S. Progress and challenges in the automated construction of Markov state models for full protein systems. *Journal of Chemical Physics* **2009**, *131*.

(S19) Pande, V. S.; Beauchamp, K.; Bowman, G. R. Everything you wanted to know about Markov State Models but were afraid to ask. *Methods* **2010**, *52*, 99–105.

(S20) Husic, B. E.; Pande, V. S. Markov State Models: From an Art to a Science. *Journal of the American Chemical Society* **2018**, *140*, 2386–2396.

(S21) Metzner, P.; Schütte, C.; Vanden-Eijnden, E. Transition Path Theory for Markov Jump Processes. *Multiscale Modeling & Simulation* **2009**, *7*, 1192–1219.

(S22) E., W.; Vanden-Eijnden, E. Towards a Theory of Transition Paths. *Journal of Statistical Physics* **2006**, *123*, 503–523.

(S23) Head, T.; Kumar, M.; Nahrstaedt, H.; Louppe, G.; Shcherbatyi, I. scikit-optimize/scikit-optimize. 2021.

(S24) Bottaro, S.; Lindorff-Larsen, K. Biophysical experiments and biomolecular simulations: A perfect match? *Science* **2018**, *361*, 355–360.

(S25) Lewis, S.; Hempel, T.; Jiménez-Luna, J.; Gastegger, M.; Xie, Y.; Foong, A. Y.; Satorras, V. G.; Abdin, O.; Veeling, B. S.; Zaporozhets, I.; others Scalable emulation of protein equilibrium ensembles with generative deep learning. *Science* **2025**, *389*, eadv9817.

(S26) Lipman, Y.; Chen, R. T.; Ben-Hamu, H.; Nickel, M.; Le, M. Flow matching for generative modeling. *arXiv preprint arXiv:2210.02747* **2022**,
